# Case report: recurrent pituitary adenoma has increased load of somatic variants

**DOI:** 10.1186/s12902-020-0493-x

**Published:** 2020-01-29

**Authors:** Raitis Peculis, Inga Balcere, Ilze Radovica-Spalvina, Ilze Konrade, Olivija Caune, Kaspars Megnis, Vita Rovite, Janis Stukens, Jurijs Nazarovs, Austra Breiksa, Aigars Kiecis, Ivars Silamikelis, Valdis Pirags, Janis Klovins

**Affiliations:** 1 Latvian Biomedical Research and Study Centre, Ratsupites str. 1-k1, Riga, LV-1067 Latvia; 2 Riga East Clinical University Hospital, Hipokrata str. 2, Riga, LV-1038 Latvia; 3 Riga Stradins University, Dzirciema str. 16, Riga, LV-1007 Latvia; 4 University of Latvia, Raina blvd. 19, Riga, LV-1586 Latvia; 5 Pauls Stradins Clinical University Hospital, Pilsonu str. 13, Riga, LV-1002 Latvia

**Keywords:** Recurrent pituitary adenoma, NFPA, Pituitary adenoma exome sequencing, Tumour variant analysis

## Abstract

**Background:**

Pituitary adenomas (PA) have an increased potential for relapse in one to 5 years after resection. In this study, we investigated the genetic differences in genomic DNA of primary and rapidly recurrent tumours in the same patient to explain the causality mechanisms of PA recurrence.

**Case presentation:**

The patient was a 69-year-old female with non-functional pituitary macroadenoma with extension into the left cavernous sinus (Knosp grade 2) who underwent craniotomy and partial resection in August 2010. Two years later, the patient had prolonged tumour growth with an essential suprasellar extension (Knosp grade 2), and a second craniotomy with partial tumour resection was performed in September 2012. In both tumours, the KI-67 level was below 1.5%. Exome sequencing via semiconductor sequencing of patient germline DNA and somatic DNA from both tumours was performed. Tmap alignment and Platypus variant calling were performed followed by variant filtering and manual review with IGV software. We observed an increased load of missense variants in the recurrent PA tumour when compared to the original tumour. The number of detected variants increased from ten to 26 and potential clonal expansion of four variants was observed. Additionally, targeted SNP analysis revealed five rare missense SNPs with a potential impact on the function of the encoded proteins.

**Conclusions:**

In this case study, an SNP located in *HRAS* is the most likely candidate inducing rapid PA progression. The relapsed PA tumour had a higher variation load and fast tumour recurrence in this patient could be caused by clonal expansion of the leftover tumour tissue.

## Background

Pituitary adenomas (PA) are neoplasms of the adenohypophyseal cells with benign characteristics but without clear markers for enhanced expansion or tumour regrowth after an operation. Clinically significant adenomas are rare and affect about one in 1000 to 1300 individuals [1, 2], although there are reports of the prevalence of up to one PA in 200 individuals [3]. Between 35 and 40% of diagnosed PAs are non-functional tumours (NFPA) that do not secrete hormones at detectable levels, while the rest (about 60–65%) are hormone-secreting PAs [4]. Remission after surgery is reported to be 35.3–44.4% for NFPAs, 60.9% for somatotroph adenomas, 72.7% for corticotroph adenomas and 61.7% for lactotroph adenomas. The highest recurrence rate has been reported at 1–5 years after surgery [5, 6].

Both genetic and epigenetic factors are involved in the development of PA [7]. However, the genetic causes of the majority of PA cases remain undiscovered, and the reason for this may be that a variety of somatic single nucleotide variants (SNVs) can trigger the development of clinically significant PA. The Knudson two-hit hypothesis is one explanation for the tumourigenesis of sporadic PA [8]. This is supported by the finding that PA has a monoclonal origin [9].

Known causal genes for sporadic PA include *GNAS*, *PI3KCA* and *USP8,* as well as known familial PA genes (such as *MEN1* and *AIP*), that often harbouring missense and nonsense SNVs in sporadic tumours [10–15].

Recently, several studies have been performed to search for novel PA variants in genomic and tumour DNA pairs using whole-exome and genome sequencing. Newey et al. in 2013 sequenced the exomes of seven NFPA and blood-derived DNA pairs. They found low levels of somatic variants, which correspond to the benign nature of PA. The average novel variant rate was 3.5 per adenoma (with a range of 1–7) and no overlapping mutated genes were found in the follow-up group consisting of 24 PA cases [16]. A study performed in the East Asian population took the systematic approach of sequencing seven types of PA including 20 NFPA samples. In that study, no overlapping NFPA variants were found, but *TRIP12* and *IARS* had variants shared with adrenocorticotropic hormone (ACTH)-secreting PA and prolactin (PRL)-secreting PA, respectively, providing insight into potential general genetic drivers of PA development. The median somatic novel variant rate per NFPA exome was 4.3 (range 0–13) [17]. A separate study identified a heterozygous missense variant c.4136G > T (p. Arg1379Leu) in cadherin-related 23 (*CDH23*) in a family with inherited PA, and the gene was later screened in familial PA, sporadic PA and healthy controls. The results revealed that four out of 12 families (33%) and 15 out of 125 (12%) sporadic PA patients had variants in *CDH23*, providing yet another candidate for the development of PA [18]. The role of potential drivers in the clonal evolution of PA cells is still to be elucidated.

Many studies of malignant tumour types have sought to gain insight into the process of carcinogenesis using massively parallel genomics to assess several tumours from the same individual (primary, secondary, relapse tumour or metastasis), revealing major intratumoural heterogeneity that guides tumour evolution [19]. It has been shown that recurrent lung adenocarcinomas display a higher proportion of sub-clonal variants (40%) compared to primary tumours without relapse (17%) [20], indicating that the overall novel variant load may increase recurrence potential. There are indications of clonal expansion in some forms of non-malignant neoplasms. For colorectal adenomas, it has been demonstrated that the same individual has heterogeneous methylation patterns in several independent tumours [21], and another study has shown a shift to malignant forms of usually non-metastatic dermatofibrosarcoma protuberans with a potential influence of therapy-induced variants and more aggressive sub-clone prevalence [22].

In this study, we used a rapid recurrence case of NFPA to gain insight into potential intratumoural genetic evolution within the same individual using exome sequencing of a tumour’s somatic DNA. The presented study is the first to report an exome scale profile of somatic variants in PA in a primary and recurrent tumour and indicates the contributing factors for PA clonal evolution and tumour relapse.

## Case presentation

The patient was a 69-year-old female who was diagnosed with pituitary macro-adenoma in 2010. At the time of diagnosis, the patient complained of a visual disturbance. The investigation revealed bi-temporal hemianopsia, *nervus opticus* right side atrophy and left side sub-atrophy.

**Fig. 1:**
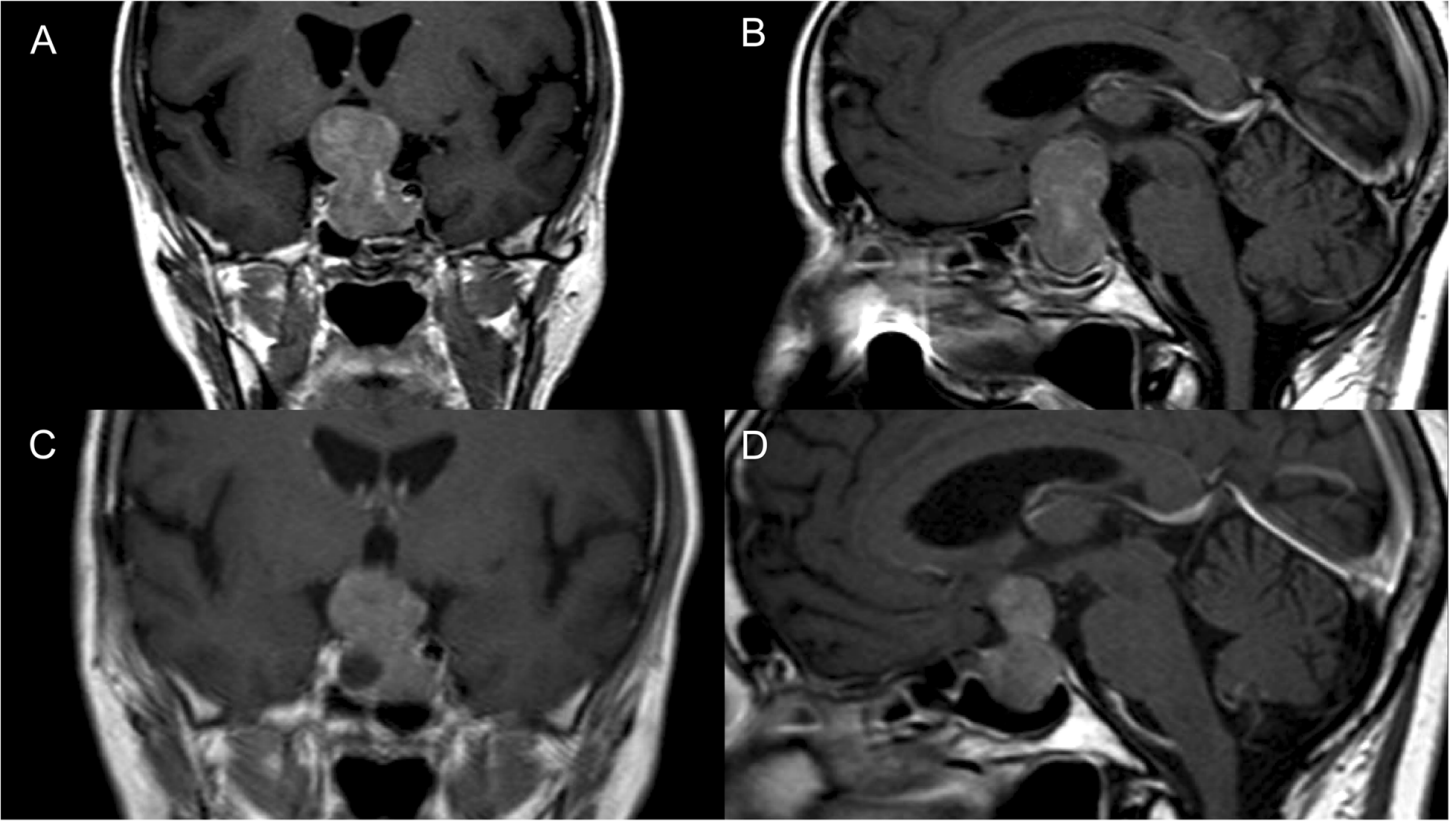
The coronal (**a**) and sagittal (**b**) magnetic resonance images of pituitary macroadenoma with suprasellar extension into the left cavernous sinus (Knosp grade 2). The images were obtained before the first surgery in July 2010. The coronal (**c**) and sagittal (**d**) magnetic resonance images of pituitary macroadenoma with prolonged tumour growth with essential suprasellar and extension into both cavernous sinuses. The images were obtained before the second surgery in July 2012

A hormonal investigation showed an NFPA. Preoperative magnetic resonance imaging (MRI) demonstrated an endo-, supra- and parasellar macroadenoma with extension into the left cavernous sinus (Knosp grade 2) with a total CC dimension of 3,86 cm and a large suprasellar component (Fig. 1). In August 2010, the patient underwent surgery via craniotomy, and partial resection of the suprasellar part of the pituitary adenoma was performed. Before the surgery, the patient was enrolled in national government-funded biobank - the Genome Database of the Latvian population (LGDB) [23]. During recruitment patient has signed two written informed consents (1) broad consent for LGDB for the use of the samples and data for health-related studies, and (2) project-specific consent for pituitary tumour studies. Both written informed consents obtained from the patient and biobank and pituitary tumour studies have been approved by the Central Medical Ethics Committee of Latvia (protocol No. 22.03.07/A7 and 01.29.1/28, respectively). Written informed consent for publication of the clinical details and/or clinical images was obtained from the patient. A copy of the consent form is available for review by the Editor of this journal.

**Fig. 2:**
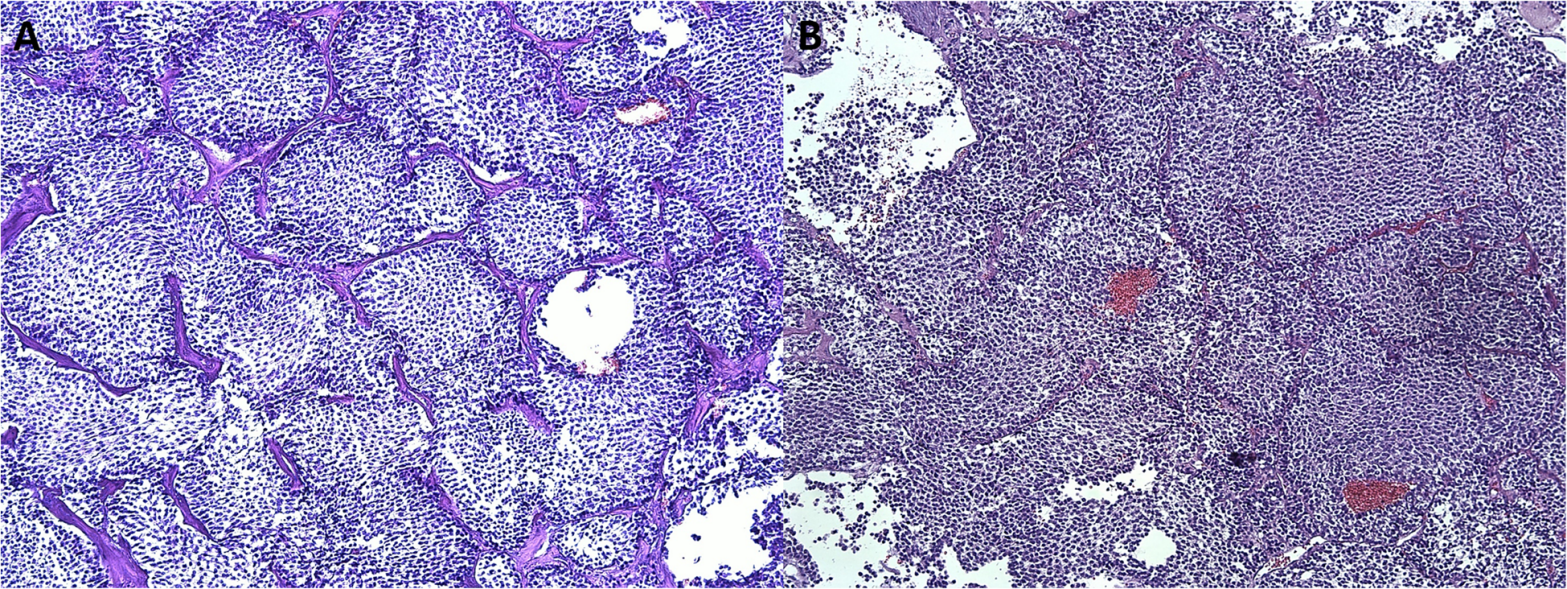
Haematoxylin and eosin staining of **a** primary and **b** recurrent formalin-fixed paraffin-embedded sections of the tumour, in 10× magnification. Both adenomas are composed of small-to-medium size chromophobic and in some places poorly basophilic, monomorphic, rounded cells with round nuclei, disperse nuclear chromatin and in some places with a well-developed nucleolus

Immunohistochemical analysis of the tumour tissue showed negative immunostaining for p53 protein expression, PRL, growth hormone (GH), ACTH, thyroid-stimulating hormone (TSH), follicle-stimulating hormone beta-subunit (FSH), somatostatin receptor 5 (SSTR5) and T-box transcription factor TBX19 (T-Pit); mild immunostaining for POU domain class 1 transcription factor 1 (PIT1); moderate immunostaining for steroidogenic factor 1 (SF1); and strong positive immunostaining for luteinizing hormone beta subunit (LH), glycoprotein hormones alpha subunit (CGA), somatostatin receptor 2 (SSTR2) and aryl hydrocarbon receptor-interacting protein (AIP). The distribution of cytokeratin-8 (CK8) was diffuse throughout the tumour, the KI-67 level was 1.5%. Haematoxylin and eosin staining of the tumour is presented in Fig. 2. The patient developed secondary hypothyroidism and secondary adrenal insufficiency after surgery what was substituted according to thyrotropic and corticotropic axis. She was started on thyroxin and hydrocortisone supplementation. The first postoperative MRI was performed 3 months after surgery. A clinical record indicates that MRI showed a large residual tumour at the left side of the cavernous sinus and a cystic structure that was also suprasellar. No images were available in the electronic medical records of the patient for this procedure. Subsequent radiotherapy was declined.

Two years later, an MRI of the pituitary gland (Fig. 1) revealed prolonged tumour growth with an essential suprasellar extension (Knosp grade 2). The patient was on thyroxin and hydrocortisone, and clinical evaluation revealed NFPA with partial hypopituitarism after the first surgery. The patient underwent a second craniotomy with partial tumour resection in September 2012. The immunohistochemistry analysis showed negative immunostaining for ACTH, TSH and T-Pit; mild immunostaining for PRL, GH, FSH, SSTR5 and PIT1; moderate immunostaining for LH, CGA and SF1; and strong positive immunostaining for SSTR2 and AIP. The distribution of CK8 was diffuse throughout the tumour, and the KI-67 level was 1%. After the second operation, the patient continued medication as before. The visual fields were unchanged when compared with 2010.

The patient was lost to follow-up, as she rarely attends her family doctor and does not want further special examination. A detailed description of additional clinical data, materials, methods and timeline following the CARE guidelines are presented in Additional file 1.

### Exome sequencing results

Final average exome (defined by UCSC Genome Browser (GRCh37/hg19) [24] interval file in Additional file 2) coverage was 60.5 ± 42.7X for white blood cells (WBC) derived DNA, 70.9 ± 210.7X for the exome of the first tumour and 84.8 ± 273.9X for the exome of the second tumour (for additional coverage, alignment and quality metrics see Additional file 3).

The results of the exome-wide search for missense and nonsense variants are shown in Table 1. In total, 26 missense and zero non-sense variants were found in the exomes from the PA tumours of this patient. Ten variants were present in the first and second tumour but not in the germline DNA derived from patients WBC. Two of those variants showed evidence for statistically significant clonal expansion after the first surgery (*C11orf57* from 5.3% (4 reads at 76X depth) to 24.3% (17 reads at 70X) and *MED24* from 3.2% (2 reads at 62X) to 22.2% (14 reads at 63X). An additional 16 variants were found in the second tumour but not in the exome of the WBC or in the exome of the first tumour. However, it should be mentioned that six (*KIAA1841*, *DMXL1*, *AK9*, *PTPRK*, *PLEKHA7* and *ITGAX*) of these 16 positions had lower than 10X coverage in the first tumour sequencing. Additionally, two genetic variants were represented in the germline DNA derived from WBC by one sequencing read of 53 (1,9%) and 70 (1,4%), respectively, and have an expanded fraction of mutant alleles in the exomes from the PA samples.

**Table 1:** Missense variants from exome sequencing of WBC and two consecutive PA derived DNA samples

	Location	Ref /Alt allele	Gene	CDS position	Protein position	Amino acids	SIFT	PolyPhen	Locus coverage/alternative variant depth		
									WBC	1st tumour	2nd tumour
Somatic variants of the both tumours	5:140712358^a^	G/A	*PCDHGA1*	2107	703	V/I	0.18	0.021	41/0	40/34 (85%)	30/12 (40%)
	11:107375677	A/T	*ALKBH8*	1702	568	C/S	0.43	0	41/0	80/27 (34%)	43/13 (30%)
	11:111951148	T/A	*C11orf57*	99	33	D/E	0	0.721	22/0	76/4 (5.3%)	70/17 (24%)
	11:62301128	C/T	*AHNAK*	761	254	G/E	0.18	0.999	94/0	64/29 (45%)	38/13 (34%)
	14:95669606^a^	A/G	*CLMN*	2080	694	C/R	0.03	0	36/0	151/51 (34%)	260/75 (29%)
	16:2855123	G/T	*PRSS41*	–	–	–	–	–	105/0	73/11 (15%)	58/14 (24%)
	17:38189695	T/G	*MED24*	631	211	I/L	0.07	0.003	26/0	62/2 (3.2%)	63/14 (22%)
	19:53384666^a^	C/A	*ZNF320*	713	238	S/I	1	0.005	55/0	9/1 (11%)	27/10 (37%)
	20:23731308	G/T	*CST1*	196	66	R/S	0	0.985	53/0	252/103 (41%)	241/125 (52%)
	X:63444842	C/G	*ASB12*	689	230	C/S	0.02	0.977	112/0	651/228 (35%)	632/234 (37%)
Somatic variants of the second tumour	2:11593766	T/C	*E2F6*	97	33	N/D	0	0.132	125/0	150/0	117/13 (11%)
	2:61349274	T/C	*KIAA1841*	2134	712	F/L	0.13	0.001	25/0	7/0	12/6 (50%)
	3:100058013	G/C	*NIT2*	90	30	E/D	0.06	0.005	22/0	34/0	38/6 (16%)
	5:112178502	T/C	*APC*	7211	2404	M/T	0.54	0	86/0	18/0	30/7 (23%)
	5:118552606	A/C	*DMXL1*	7937	2646	D/A	0	0.234	22/0	4/0	6/5 (83%)
	5:176797955	A/C	*RGS14*	1177	393	T/P	0	0.962	1/0	15/0	42/10 (24%)
	6:109480584	A/C	*CEP57L1*	935	312	D/A	0.88	0.08	30/0	11/0	20/6 (30%)
	6:109983834	G/T	*AK9*	364	122	Q/K	0.06	0.738	37/0	1/0	69/19 (28%)
	6:128326254	C/T	*PTPRK*	2499	833	M/I	0.02	0.024	90/0	3/0	16/7 (44%)
	8:17417949	A/G	*SLC7A2*	1411	471	R/G	0.41	0	133/0	13/0	33/8 (24%)
	9:123751971	G/A	*C5*	3029	1010	A/V	0.21	0.713	75/0	25/0	23/8 (35%)
	10:120801963	A/C	*EIF3A*	3069	1023	D/E	0.12	0.005	75/0	21/0	43/15 (35%)
	11:16812380	C/T	*PLEKHA7*	3017	1006	G/D	0.12	0.575	57/0	8/0	32/6 (19%)
	11:77911754	G/A	*USP35*	1097	366	G/E	0.02	0.212	74/0	13/0	20/7 (35%)
	12:113730866	G/A	*TPCN1*	2457	819	M/I	0.17	0.053	88/0	70/0	25/10 (40%)
	16:31371749	G/C	*ITGAX*	826	276	A/P	0	0.999	15/0	7/0	12/7 (58%)
Variants with expansion	4:85707175	C/T	*WDFY3*	4019	1340	R/Q	0	0.787	53/1 (1.9%)	37/14 (38%)	23/10 (43%)
	6:132171199	A/G	*ENPP1*	383	128	E/G	0.38	0.003	70/1 (1.4%)	75/2 (2.7%)	34/10 (29%)

^a^Cosmic database variant (5:140712358, *PCDHGA1,* 703 V/I, COSV65295567; 14:95669606, *CLMN,* 694C/R, COSV54187254; 19:53384666, *ZNF320,* 238S/I, COSV67089305)

Gene ontology analysis revealed that three of the genes are associated with the RNA binding process and two of them, *ALKBH8* and *AHNAK*, contain a variant in the first tumour. *RGS14* and *C5* are involved in the G protein alpha signalling pathway, and *APC* and *PCDHGA1* are involved in the Wnt signalling pathway, but the products of *ENPP1* and *PTPRK* possess phosphatase activity. Three variants have been previously reported in COSMIC database *PCDHGA1* (COSV65295567) in three cases - one prostate, one endometrium and one large intestine tumours. *CLMN* (COSV54187254) has been reported in one cervical cancer sample and *ZNF320* (COSV67089305) in one thyroid carcinoma sample.

**Fig. 3:**
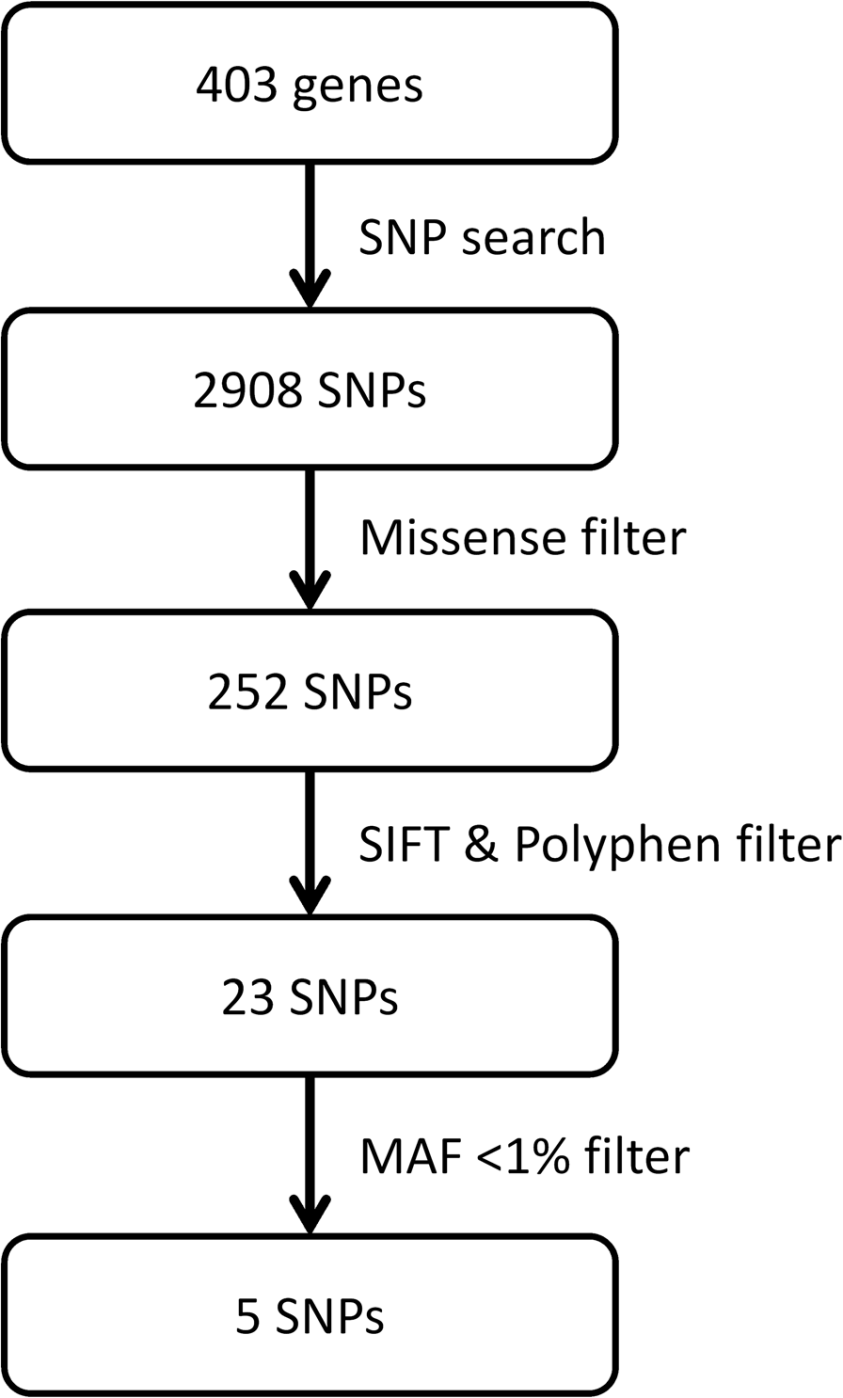
Targeted SNP search and filtering steps

We also performed a targeted analysis of SNPs located in a gene set compiled from the literature on PA genetics, tumour suppression and genome and exome sequencing. By searching the literature with the keywords “pituitary adenoma genetics”, “pituitary adenoma exome sequencing”, “pituitary adenoma gwas” and “tumour suppressor genes” on 15th of May 2018, a list of 403 genes was compiled (Additional file 4). Targeted SNP analysis was performed in this region which encompasses 47,370,914 base pairs (including introns, which are sparsely represented in exome data) (Fig. 3). Two hundred fifty-two missense SNPs were found in 133 genes. SIFT and Polyphen prediction algorithms rated 23 missense SNPs both “deleterious” and “probably damaging” or “possibly damaging”. *CDH23* was the only gene that has two SNPs (rs45583140, rs1227049) recognized by both algorithms as severely affecting protein function.

A frequency filter with minor allele frequency threshold 1% (gnomAD database, non-Finnish European population) left five SNPs as the top candidates possibly contributing towards characteristics and development of PA (Table 2). Four SNPs had “start-loss”, “stop-gain” and “stop-loss” consequences, but all have high population frequencies. CNVs detection using CoNIFER v0.2.2 [25] yielded negative results.

**Table 2:** Candidate variants from targeted SNP analysis

Locus	Gene	Minor allele	Aa change	SNP code	SIFT	PolyPhen	gnomAD NFE AF
chr 14:68274264	*ZFYVE26*	T	246 A/D	rs367929006	del (0)	possibly dmg (0.79)	0
chr 20:42788571	*JPH2*	C	286 T/A	rs144022614	del (0)	possibly dmg (0.45)	0.14%
chr11:56380836	*OR5M1*	T	48 L/Q	rs183262731	del (0)	probably dmg (1)	0.55%
chr22:39222627	*NPTXR*	A	326 R/W	rs34637063	del (0)	possibly dmg (0.83)	0.59%
chr11:533515	*HRAS*	A	130 A/S	COSV54248437	del (0.04)	possibly dmg (0.71)	–
chr10:73571765^a^	*CDH23*	C	3130 F/L	rs45583140/ COSV56481145	del (0.01)	probably dmg (0.99)	3.6%
10:73434888^a^	*CDH23*	C	495 G/A	rs1227049	del (0)	probably dmg (1)	17.2%

*Aa* amino acid, *SNP* single nucleotide polymorphism, *NFE* non-Finnish European, *AF* allele frequency, *del* deleterious, *dmg* damaging

^a^compound population frequency 0.6%

## Discussion and conclusions

Here we present the first study performing exome sequencing of recurrent PA from the same patient. Although the remission rate of NFPA after surgery is 44,4% [5], to the best of our knowledge, exome or genome analysis of a recurrent pituitary tumour pair is not yet published. At the same time, there are studies on clonal expansion and intra-tumoural heterogeneity performed for a multitude of other tumour types [19, 20, 22]. Our results indicate that rapid recurrence of PA is led by an increase of novel variant load in a benign tumour that might be explained by clonal selection of leftover tumour tissue. We also would like to notice that the patient did not receive radiotherapy during any stage of the treatment that would stimulate additional mutagenesis.

The number of variants in primary PA is low, is consistent with the literature reported in other exome sequencing studies on PA [16, 26] and corresponds to the mainly non-metastatic phenotype of PA. In spite of the relatively benign nature of PA, the regrowth in 2 years after the first surgery indicates the proliferative potential of the tumour cells in the studied patient. Previous epidemiological studies have shown that recurrence of PA is most likely to happen during the 5 year period after surgery [5]. Our study indicates that a large proportion of SNVs from the first tumour was also present in the second tumour. This might indicate that, at least in this patient, regrowth occurred from leftover cells that were missed when resecting the primary tumour. Whether this finding can be generalized for all recurrent PAs should be explored with more extensive studies. We also observed an increased missense variant burden in the second PA when compared to the first tumour. This increase in variant content can be explained by a diminished ability to control DNA replication or influence of some of the SNVs that can serve as “drivers” for clonal expansion when novel “passenger” variants are acquired. This has been observed frequently in other malignant cancers [27, 28], and there is some indication in similar processes in more neutral tumours [22], but this aspect has not been widely studied in benign tumours. Alternatively, if not all of the PA tumour mass was removed during the first surgery (in this particular case, leftover tissue was detected during an MRI scan 3 months after surgery), it implies that either the original tumour was not monoclonal, or the accumulation of variants might have led to the development of a group of heterogeneous clones. However, the monoclonality of PA has been widely accepted [9, 16, 29]. Shared variants between the tumour samples in our study also provide a high likelihood of PA recurrence rather than the development of a separate novel neoplasm. Additionally, we did not observe any cell lineage change or significant alteration of hormone production in presented patient’s case. There are reports that in rare cases shift from clinically silent PA to hormonally active tumours is observed [30–32], however, these do not include exact tracing according to transcription factors defined by the WHO classification of 2017. To further assess potential shifts in cell lineage of operated recurrent PA cases comprehensive research of significant number of relapse PAs including T-pit, SF1, PIT-1 immuno-staining is needed.

It is unlikely that the variants identified are the cause of the tumours either alone or in a compound fashion, and the most likely explanation is that these are somatic “passenger” variants. Four genes (*WDFY3*, *ENPP1*, *C11orf57* and *MED24*) with a low amount of the mutated reads in the germline DNA or the first tumour could be attributed to the allele-specific replication of the germline and/or the first tumour during library preparation.

The increased number of variants in the second tumour could indicate a decreased ability of the tumour cells to repair DNA damage as proliferation increases and variants accumulate. It could also show that the tumour is transforming or being transformed by surgery towards a relatively hypermutated state with multiple disruptions contributing to the aggressiveness of the tumour. Although this “hypermutated” PA state is lower than the novel variant rate in hypermutated malignant tumours [33], it is significantly more than the average novel variant rate per PA exome of other studies [16, 17, 34].

A targeted study of genes compiled from the literature on PA genetics and tumourigenesis revealed several interesting facts. *CDH23* contained two SNPs (rs45583140, MAF in non-Finnish Europeans = 3.6%, rs1227049, MAF in non-Finnish Europeans = 17.2%) that are missense and predicted to be damaging to the protein function. *CDH23* encodes cadherin 23, which is involved in cell to cell adhesion [35] and hearing mechanism [36]. Cadherin 23 is localized at cell contact points, suggesting involvement in contact inhibition, and it is highly expressed in breast cancer compared to normal tissue [35]. Additionally, the *CDH23* variant p.Arg1379Leu was found in a PA-affected family, and further screening showed that 33% of familial PA patients and 125 of sporadic PA patients carry functional variants compared to 0.8% of controls [18]. Therefore, it is possible that the two identified compound heterozygous *CDH23* missense SNPs (a population chance of 0.6%) could contribute to the quick PA regrowth in the patient studied via disrupted contact inhibition.

The five other SNPs identified in the targeted gene search are located in *ZFYVE26, JPH2, OR5M1, NPTXR* and *HRAS*. All SNPs were in the heterozygous state. Three of the genes JPH2, OR5M1 and NPTXR have had a somatic variant (although different position) in previous PA exome sequencing studies in the literature [26, 37], and further functional studies are needed to evaluate the role of these gene variants on pituitary cell functionality. Recurrence of deleterious rare SNVs across different PA sequencing studies could indicate that they could be part of Knudson’s two hit hypothesis in PA, especially when taking into account other genetic and/ or environmental factors. ZFYVE26 has been shown to harbour somatic mutations in hereditary non- polyposis colorectal cancer and is expressed in hepatocellular adenocarcinoma) [38], but the potential relation to PA development is discussable. HRAS is widely described proto-oncogene which predominates in head and neck squamous cell carcinoma [39] but also is found in the salivary duct [40], bladder urothelial carcinoma and acute myeloid leukaemia [39]. Having been implicated in other carcinogenesis cases, *HRAS* could be a novel candidate that influences PA. A cosmic somatic variant (COSV54248437) found in our PA patient has been previously found in cervix cancer [41]. Together with both prediction algorithms assigning a negative impact on protein functionality and the virtual absence in the general population, this could be one of the prime candidates that causes development or rapid growth of the tumour in our study.

This study has some limitations. One limitation is that exome sequencing may miss important variants outside the coding regions that may contribute to PA development. Whole-genome sequencing would overcome this limitation. Similarly, although we had good sequencing coverage in our samples, increased depth would provide more precision for variants identified in a low percentage of reads. Additional sequencing, however, is not possible due to the low amount of initial tumour material available and the division of this material for the clinical archive and other research activities. We also did not perform Sanger sequencing validation as in other cancer studies where the overall variant percentage in the tumour somatic material could be low, as these may not be detectable by Sanger sequencing [17, 26, 37].

In conclusion, we show that in this relapse case, the regrowth of PA is accompanied by the increase of the tumour novel variant load, which could be caused by clonal expansion of the tumour leftover tissue.

## Supplementary information

**Additional file 1.** Timeline following the CARE guidelines. Additional clinical data. A detailed description of Materials and Methods used in the study.

**Additional file 2.** Interval file of HG19 exome coordinates. This reference exome version was used to search for variants and CNVs.

**Additional file 3.** Document file (30 pages) describing alignment quality metrics for each sample (10 pages per sample).

**Additional file 4.** Excel spreadsheet with the list of genes used for targeted PA genetic marker search.

## Data Availability

The datasets used and/or analysed during the current study are available from the corresponding author on reasonable request.

## Abbreviations

ACTH: Adrenocorticotropic hormone
DNA: Deoxyribonucleic acid
IGV: Integrative genomics viewer
LGDB: Genome Database of Latvian population
MAF: Minor allele frequency
MRI: Magnetic resonance imaging
NFPA: Non-functional pituitary adenoma
PA: Pituitary adenoma
RNA: Ribonucleic acid
SNP: Single nucleotide polymorphism
SNV: Single nucleotide variant
WBC: White blood cell

## References

[1] Daly AF, Rixhon M, Adam C, Dempegioti A, Tichomirowa MA, Beckers A (2006). High prevalence of pituitary adenomas: a cross-sectional study in the province of Liege, Belgium. J Clin Endocrinol Metab.

[2] Fernandez A, Karavitaki N, Wass JA (2010). Prevalence of pituitary adenomas: a community-based, cross-sectional study in Banbury (Oxfordshire, UK). Clin Endocrinol.

[3] Rosario PW (2011). Frequency of acromegaly in adults with diabetes or glucose intolerance and estimated prevalence in the general population. Pituitary.

[4] Famini P, Maya MM, Melmed S (2011). Pituitary magnetic resonance imaging for sellar and parasellar masses: ten-year experience in 2598 patients. J Clin Endocrinol Metab.

[5] Roelfsema F, Biermasz NR, Pereira AM (2012). Clinical factors involved in the recurrence of pituitary adenomas after surgical remission: a structured review and meta-analysis. Pituitary.

[6] Tampourlou M, Ntali G, Ahmed S, Arlt W, Ayuk J, Byrne JV (2017). Outcome of nonfunctioning pituitary adenomas that regrow after primary treatment: a study from two large UK centers. J Clin Endocrinol Metab.

[7] Peltomaki P (2012). Mutations and epimutations in the origin of cancer. Exp Cell Res.

[8] Knudson AG (1993). Antioncogenes and human cancer. Proc Natl Acad Sci U S A.

[9] Jacoby LB, Hedley-Whyte ET, Pulaski K, Seizinger BR, Martuza RL (1990). Clonal origin of pituitary adenomas. J Neurosurg.

[10] Lin Y, Jiang X, Shen Y, Li M, Ma H, Xing M (2009). Frequent mutations and amplifications of the PIK3CA gene in pituitary tumors. Endocr Relat Cancer.

[11] Murat CB, Braga PB, Fortes MA, Bronstein MD, Correa-Giannella ML, Giorgi RR (2012). Mutation and genomic amplification of the PIK3CA proto-oncogene in pituitary adenomas. Braz J Med Biol Res.

[12] Reincke M, Sbiera S, Hayakawa A, Theodoropoulou M, Osswald A, Beuschlein F (2015). Mutations in the deubiquitinase gene USP8 cause Cushing's disease. Nat Genet.

[13] Shi Y, Tang D, Deng J, Su C (1998). Detection of gsp oncogene in growth hormone-secreting pituitary adenomas and the study of clinical characteristics of acromegalic patients with gsp-positive pituitary tumors. Chin Med J.

[14] Taboada GF, Tabet AL, Naves LA, de Carvalho DP, Gadelha MR (2009). Prevalence of gsp oncogene in somatotropinomas and clinically non-functioning pituitary adenomas: our experience. Pituitary.

[15] Peculis R, Balcere I, Rovite V, Megnis K, Valtere A, Stukens J (2016). Polymorphisms in MEN1 and DRD2 genes are associated with the occurrence and characteristics of pituitary adenomas. Eur J Endocrinol.

[16] Newey PJ, Nesbit MA, Rimmer AJ, Head RA, Gorvin CM, Attar M (2013). Whole-exome sequencing studies of nonfunctioning pituitary adenomas. J Clin Endocrinol Metab.

[17] Song ZJ, Reitman ZJ, Ma ZY, Chen JH, Zhang QL, Shou XF (2016). The genome-wide mutational landscape of pituitary adenomas. Cell Res.

[18] Zhang Q, Peng C, Song J, Zhang Y, Chen J, Song Z (2017). Germline mutations in CDH23, encoding cadherin-related 23, are associated with both familial and sporadic pituitary adenomas. Am J Hum Genet.

[19] McGranahan N, Swanton C (2017). Clonal heterogeneity and tumor evolution: past, present, and the future. Cell.

[20] Zhang J, Fujimoto J, Zhang J, Wedge DC, Song X, Zhang J (2014). Intratumor heterogeneity in localized lung adenocarcinomas delineated by multiregion sequencing. Science.

[21] Humphries A, Cereser B, Gay LJ, Miller DS, Das B, Gutteridge A (2013). Lineage tracing reveals multipotent stem cells maintain human adenomas and the pattern of clonal expansion in tumor evolution. Proc Natl Acad Sci U S A.

[22] Oh E, Jeong HM, Kwon MJ, Ha SY, Park HK, Song JY (2017). Unforeseen clonal evolution of tumor cell population in recurrent and metastatic dermatofibrosarcoma protuberans. PLoS One.

[23] Rovite Vita, Wolff-Sagi Yael, Zaharenko Linda, Nikitina-Zake Liene, Grens Elmars, Klovins Janis (2018). Genome Database of the Latvian Population (LGDB): Design, Goals, and Primary Results. Journal of Epidemiology.

[24] Kent WJ, Sugnet CW, Furey TS, Roskin KM, Pringle TH, Zahler AM (2002). The human genome browser at UCSC. Genome Res.

[25] Krumm N, Sudmant PH, Ko A, O'Roak BJ, Malig M, Coe BP (2012). Copy number variation detection and genotyping from exome sequence data. Genome Res.

[26] Bi WL, Horowitz P, Greenwald NF, Abedalthagafi M, Agarwalla PK, Gibson WJ (2017). Landscape of genomic alterations in pituitary adenomas. Clin Cancer Res.

[27] Kim KP, Kim JE, Hong YS, Ahn SM, Chun SM, Hong SM (2017). Paired primary and metastatic tumor analysis of somatic mutations in synchronous and Metachronous colorectal Cancer. Cancer Res Treat.

[28] Xie F, Zhang Y, Mao X, Zheng X, Han-Zhang H, Ye J (2018). Comparison of genetic profiles among primary lung tumor, metastatic lymph nodes and circulating tumor DNA in treatment-naive advanced non-squamous non-small cell lung cancer patients. Lung Cancer.

[29] Ibanez-Costa A, Korbonits M (2017). AIP and the somatostatin system in pituitary tumours. J Endocrinol.

[30] Andino-Rios GG, Portocarrero-Ortiz L, Rojas-Guerrero C, Terrones-Lozano A, Ortiz-Plata A, Reza-Albarran AA (2018). Nonfunctioning pituitary adenoma that changed to a functional Gonadotropinoma. Case Rep Endocrinol.

[31] Daems T, Verhelst J, Michotte A, Abrams P, De Ridder D, Abs R (2009). Modification of hormonal secretion in clinically silent pituitary adenomas. Pituitary.

[32] Lania AG, Ferrero S, Pivonello R, Mantovani G, Peverelli E, Di Sarno A (2010). Evolution of an aggressive prolactinoma into a growth hormone secreting pituitary tumor coincident with GNAS gene mutation. J Clin Endocrinol Metab.

[33] Campbell BB, Light N, Fabrizio D, Zatzman M, Fuligni F, de Borja R (2017). Comprehensive analysis of Hypermutation in human Cancer. Cell.

[34] Valimaki N, Demir H, Pitkanen E, Kaasinen E, Karppinen A, Kivipelto L (2015). Whole-genome sequencing of growth hormone (GH)-secreting pituitary adenomas. J Clin Endocrinol Metab.

[35] Apostolopoulou M, Ligon L (2012). Cadherin-23 mediates heterotypic cell-cell adhesion between breast cancer epithelial cells and fibroblasts. PLoS One.

[36] Siemens J, Lillo C, Dumont RA, Reynolds A, Williams DS, Gillespie PG (2004). Cadherin 23 is a component of the tip link in hair-cell stereocilia. Nature.

[37] Ronchi CL, Peverelli E, Herterich S, Weigand I, Mantovani G, Schwarzmayr T (2016). Landscape of somatic mutations in sporadic GH-secreting pituitary adenomas. Eur J Endocrinol.

[38] Yu L, Yin B, Qu K, Li J, Jin Q, Liu L (2018). Screening for susceptibility genes in hereditary non-polyposis colorectal cancer. Oncol Lett.

[39] Hobbs GA, Der CJ, Rossman KL (2016). RAS isoforms and mutations in cancer at a glance. J Cell Sci.

[40] Chiosea SI, Miller M, Seethala RR (2014). HRAS mutations in epithelial-myoepithelial carcinoma. Head Neck Pathol.

[41] Zerbino DR, Achuthan P, Akanni W, Amode MR, Barrell D, Bhai J (2018). Ensembl 2018. Nucleic Acids Res.

