# Supplementary material for: Case report: recurrent pituitary adenoma has increased load of somatic variants
Source: BMC Endocr Disord. 2020 Jan 29;20:17. doi: 10.1186/s12902-020-0493-x (PMC6988340; doi:10.1186/s12902-020-0493-x)
Supplement: Supplementary file 1 — Additional file 1. Timeline following the CARE guidelines. Additional clinical data. A detailed description of Materials and Methods used in the study. [file 12902_2020_493_MOESM1_ESM.doc]

**Additional file 1.**

**Timeline following the CARE guidelines**

**
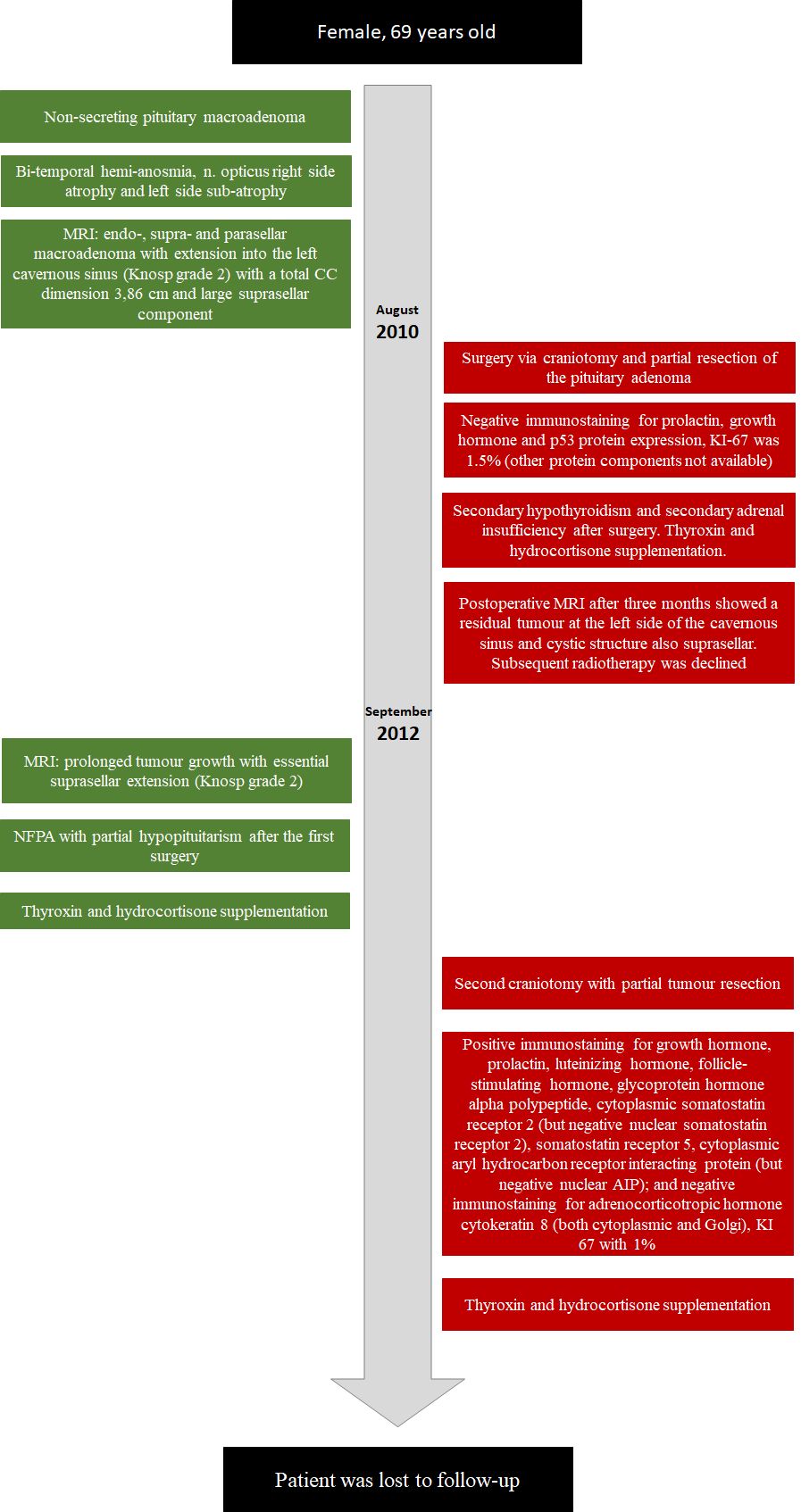
**

**Patients hormonal profile. Blood diagnostics.**

| Hormone | Reference interval | 1^st^ surgery | | 2^nd^ surgery | |
| --- | --- | --- | --- | --- | --- |
|  |  | Before | After | Before | After |
| Prolactin | 59-619 mU/L | 101 | nd | 90 | nd |
| ACTH | 0-46 pg/ml | 10.2 | nd | 8.4 | nd |
| Cortisol | 4.3-22.4 mcg/dl | 3.48 | 0.9 | 0.7 | 0.53 |
| TSH | 0.4-4.0 mcIU/ml | 1.54 | nd | 5.9 | nd |
| FT4 | 0.8-1.9 ng/dl | 0.9 | 0.76 | 0.54 | 0.51 |
| FT3 | 1.8-4.2 pg/ml | nd | 1.0 | 2.0 | 1.3 |

nd – no data available

**The coronal ( 1) and sagittal (2) magnetic resonance images of pituitary macroadenoma with parcial resection of the suprasellar part of tumour 1 year after second surgery.** *(Due to image quality this is not included in main manuscript)*

**
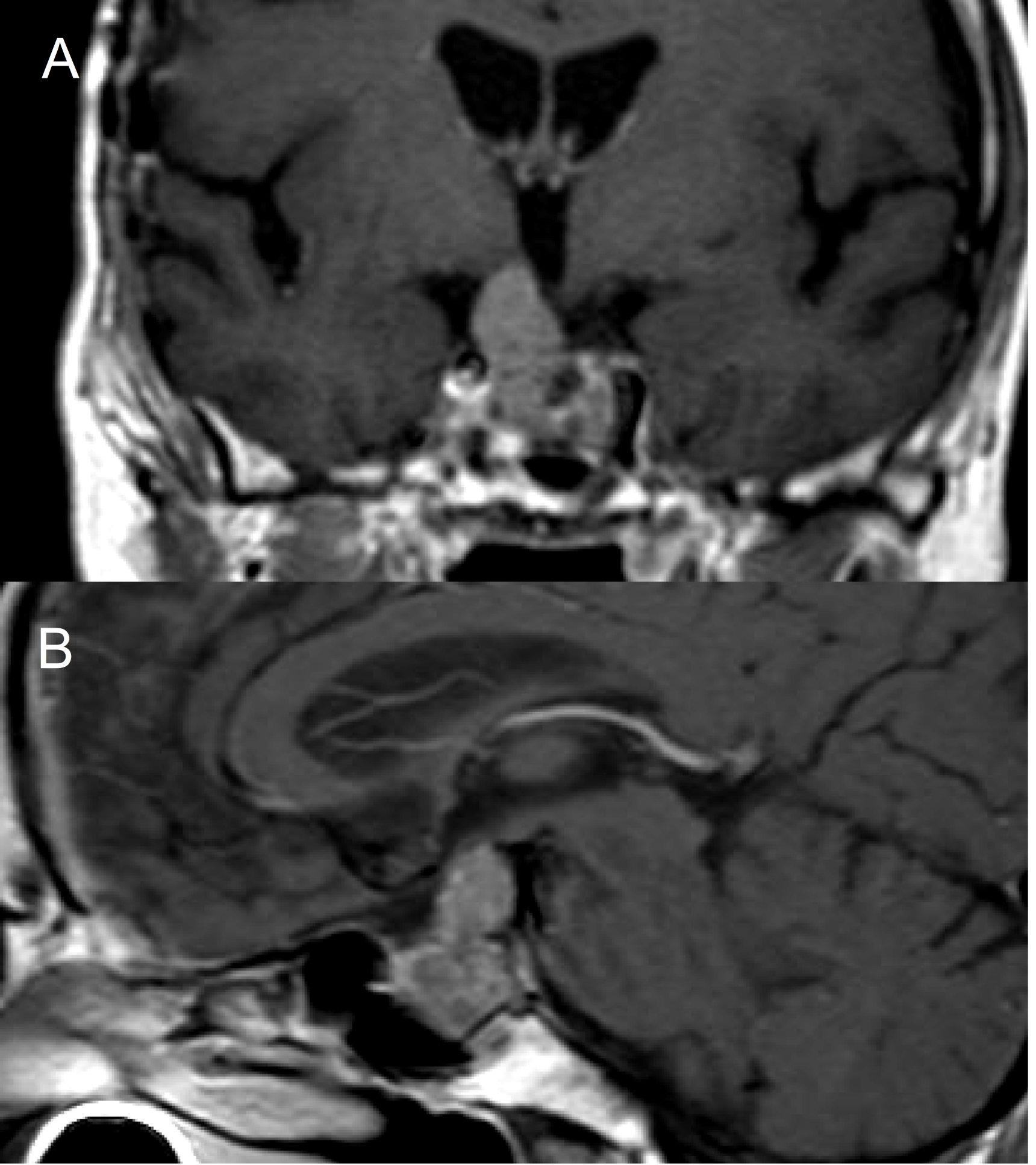
**

**Materials and Methods**

*Biological samples and data*

The patient was recruited to the Genome Database of Latvian population (LGDB), a government-funded national biobank, by an endocrinologist at Pauls Stradins Clinical University Hospital in 2010. LGDB is a central institution for human genetic material biobanking for research needs in Latvia .

Two consents were obtained from patient, broad consent for LGDB for use of biological material and medical data for human health and hereditary research, and project-specific consent with approval of the use of biological material and clinical data in research related to the pituitary tumours. Both biobank study design and PA research study have been approved by Central Medical Ethics Committee of Latvia (protocol No. 22.03.07/A7 and 01.29.1/28, respectively). Sample collection and research process comply with the Declaration of Helsinki (in particular parts A and B) and Declaration of Taipei.

*DNA preparation*

White blood cell (WBC) derived DNA was acquired from LGDB where it is prepared according to a standard protocol .

Tissue samples were homogenized using Lysing/Binding Buffer from the mirVana™ miRNA Isolation Kit (Thermo Fisher Scientific, USA), Lysing Matrix D and FastPrep®-24 (MP Biomedicals, USA). Homogenized samples were separated into phases using Homogenate Additive from the mirVana™ miRNA Isolation Kit (Thermo Fisher Scientific, USA) and ChCl_3_ (Sigma-Aldrich, USA). DNA and protein containing middle and lower phases were used for DNA extraction using precipitation method, with Na_3_C_6_H_5_O_7_ (BDH Prolabo, UK) and 10 % C_2_H_5_OH (Jaunpagasts Plus, Latvia) solution and 8mM NaOH (AppliChem, USA). Extracted DNA was purified by additional precipitation with C_6_H_5_OH (Thermo Fisher Scientific, USA) and ChCl_3_ (Sigma-Aldrich, USA).

*Library preparation*

For each sample, 1 µg of DNA was fragmented using Covaris S220 (average size 250bp). Fragment libraries were produced according to the protocol using Ion Xpress Barcode Adapters and Ion Plus Fragment Library kits. Size selection was performed using 2% DF Marker M1 agarose gel cassettes on BluePipin (target fragment length 350bp). 500ng of each library was used for exome capture using Ion TargetSeq™ Exome Kit according to the manufacturer's protocol. Agilent Bioanalyzer 2100 was used to quantify libraries with Agilent High Sensitivity DNA kit.

*Sequencing*

Manufacturer's protocols of Ion PI Template OT2 200 Kit v2, Ion PI Sequencing 200 Kit v2 and Ion PI™ Chip was used for template preparation and sequencing on Ion Proton semiconductor sequencing machine. Both somatic tissues derived DNA samples were sequenced in parallel on four chips, WBC derived DNA was sequenced on two chips: once in parallel with both somatic DNA samples and once on the separate PI chip.

*Data analysis*

Sequence reads produced by Ion Proton were aligned to the human HG19 reference with the TMAP3.4.1 software using default parameters. BAM files were merged using BAMTools, duplicate reads marked and removed using PicardTools v1.131, variants called with Platypus v 0.8.1. Variant annotation was performed using ANNOVAR. Vcf files annotated by ANNOVAR were filtered to obtain missense mutations for each sample, then merged to obtain overlapping information about all positions in all three samples where at least one sample contains missense mutation in at least 10X coverage at the position/region.

After filtering for missense and nonsense variants, only variants found in one or both adenomas were retained and manually reviewed using IGV. BAM statistics were obtained with Qualimap v2.2.1. Short (<10bp) indels were not evaluated due to sequencing technology constraints at the homopolymer sites.

CNVs were called using CoNIFER v0.2.2 (PMID: 22585873) in all three samples simultaneously from alignment (BAM) files and setting SVD to 1 (PMID: 28852425).
