# Supplementary material for: Case report: recurrent pituitary adenoma has increased load of somatic variants
Source: BMC Endocr Disord. 2020 Jan 29;20:17. doi: 10.1186/s12902-020-0493-x (PMC6988340; doi:10.1186/s12902-020-0493-x)
Supplement: Supplementary file 3 — Additional file 3. Document file (30 pages) describing alignment quality metrics for each sample (10 pages per sample). [file 12902_2020_493_MOESM3_ESM.pdf]

Qualimap Report: WBC DNA alignment quality metrics

QualiMap

CONTENTS

- Input data & parameters
- Summary
- Coverage across reference
- Coverage Histogram
- Coverage Histogram (0-50X)
- Genome Fraction Coverage
- Duplication Rate Histogram
- Mapped Reads Nucleotide Content
- Mapped Reads GC-content Distribution
- Mapped Reads Clipping Profile
- Homopolymer Indels
- Mapping Quality Across Reference
- Mapping Quality Histogram

Input data and parameters

QualiMap command line

qualimap bamqc -bam E:\NGS\Adenomas\Recidivs_5_31_155D8\155D8_gal.bam -gff E:\NGS \Exome_hg19_6fields.bed -os -c -nw 400 -hm 3

Alignment

Command line:	mapall -f ucsc.hg19.fasta -r 155D8.fastq -i fastq -s 155D8.bam -o 1 stage1 map1 stage2 map2 stage3 map3 stage4 map4
Draw chromosome limits:	yes
Analyze overlapping paired-end reads:	no
Program:	tmap (3.4.1)
Analysis date:	Mon Nov 07 18:00:20 EET 2016
Size of a homopolymer:	3
Skip duplicate alignments:	no
Number of windows:	400
BAM file:	E:\NGS\Adenomas \Recidivs_5_31_155D8\155D8_gal.bam

GFF region

Library protocol:	non-strand-specific
Outside statistics:	yes
GFF file:	E:\NGS\Exome_hg19_6fields.bed

Summary

Globals

Reference size	3 137 161 264
Number of reads	96 701 200
Mapped reads	95 113 193 / 98,36%
Unmapped reads	1 588 007 / 1,64%
Mapped paired reads	0 / 0%
Read min/max/mean length	25 / 390 / 118,23
Clipped reads	14 057 475 / 14,54%

Globals (inside of regions)

Regions size/percentage of reference	37 259 743 / 1,19%
Mapped reads	27 316 044 / 28,25%
Duplicated reads (estimated)	9 652 150 / 35,34%

Duplication rate	32,2%

ACGT Content (inside of regions)

Number/percentage of A's	547 451 009 / 24,51%
Number/percentage of C's	570 991 436 / 25,56%
Number/percentage of T's	543 068 252 / 24,31%
Number/percentage of G's	572 329 489 / 25,62%
Number/percentage of N's	0 / 0%
GC Percentage	51,18%

Coverage (inside of regions)

Mean	60,5114
Standard Deviation	42,7392

Mapping Quality (inside of regions)

Mean Mapping Quality	65,71

Mismatches and indels (inside of regions)

General error rate	4,27%
Mismatches	49 595 641
Insertions	38 336 007
Mapped reads with at least one insertion	18,27%
Deletions	29 602 103
Mapped reads with at least one deletion	16,11%
Homopolymer indels	48,32%

Chromosome stats (inside of regions)

Name	Length	Mapped bases	Mean coverage	Standard deviation
chrM	0	0	0	0
chr1	3784540	236578358	62,5118	45,2718
chr2	2784129	166139593	59,6738	37,9589
chr3	2096186	127953961	61,0413	36,9576
chr4	1419494	83387280	58,7444	35,5686
chr5	1707093	102811806	60,2262	38,9277
chr6	1939714	103559933	53,3893	38,6697
chr7	1715393	102074252	59,5049	42,1879
chr8	1223260	68815048	56,2555	37,4744
chr9	1591797	93250318	58,5818	40,5575
chr10	1501994	91620244	60,9991	39,3892
chr11	2152291	127017423	59,015	38,1026
chr12	1910362	114846390	60,1176	37,5286

chr13	706110	40270904	57,0321	35,8253
chr14	1194533	72228617	60,466	40,9563
chr15	1229578	74199915	60,3458	39,3183
chr16	1502270	86273286	57,4286	41,4636
chr17	2108732	124654331	59,1134	40,2748
chr18	566305	34395766	60,7372	40,1749
chr19	2381241	126325815	53,0504	41,9467
chr20	922966	52746968	57,1494	39,0903
chr21	401819	23057163	57,382	41,3489
chr22	836825	48488562	57,9435	42,7852
chrX	1493772	152304063	101,9594	73,0393
chrY	89339	1639888	18,3558	31,9905
chr1_gl000191_random	0	0	0	0
chr1_gl000192_random	0	0	0	0
chr4_ctg9_hap1	0	0	0	0
chr4_gl000193_random	0	0	0	0
chr4_gl000194_random	0	0	0	0
chr6_apd_hap1	0	0	0	0
chr6_cox_hap2	0	0	0	0
chr6_dbb_hap3	0	0	0	0
chr6_mann_hap4	0	0	0	0
chr6_mcf_hap5	0	0	0	0
chr6_qbl_hap6	0	0	0	0
chr6_ssto_hap7	0	0	0	0
chr7_gl000195_random	0	0	0	0
chr8_gl000196_random	0	0	0	0
chr8_gl000197_random	0	0	0	0
chr9_gl000198_random	0	0	0	0
chr9_gl000199_random	0	0	0	0
chr9_gl000200_random	0	0	0	0
chr9_gl000201_random	0	0	0	0
chr11_gl000202_random	0	0	0	0
chr17_ctg5_hap1	0	0	0	0
chr17_gl000203_random	0	0	0	0
chr17_gl000204_random	0	0	0	0
chr17_gl000205_random	0	0	0	0
chr17_gl000206_random	0	0	0	0
chr18_gl000207_random	0	0	0	0

chr19_gl000208_random	0	0	0	0
chr19_gl000209_random	0	0	0	0
chr21_gl000210_random	0	0	0	0
chrUn_gl000211	0	0	0	0
chrUn_gl000212	0	0	0	0
chrUn_gl000213	0	0	0	0
chrUn_gl000214	0	0	0	0
chrUn_gl000215	0	0	0	0
chrUn_gl000216	0	0	0	0
chrUn_gl000217	0	0	0	0
chrUn_gl000218	0	0	0	0
chrUn_gl000219	0	0	0	0
chrUn_gl000220	0	0	0	0
chrUn_gl000221	0	0	0	0
chrUn_gl000222	0	0	0	0
chrUn_gl000223	0	0	0	0
chrUn_gl000224	0	0	0	0
chrUn_gl000225	0	0	0	0
chrUn_gl000226	0	0	0	0
chrUn_gl000227	0	0	0	0
chrUn_gl000228	0	0	0	0
chrUn_gl000229	0	0	0	0
chrUn_gl000230	0	0	0	0
chrUn_gl000231	0	0	0	0
chrUn_gl000232	0	0	0	0
chrUn_gl000233	0	0	0	0
chrUn_gl000234	0	0	0	0
chrUn_gl000235	0	0	0	0
chrUn_gl000236	0	0	0	0
chrUn_gl000237	0	0	0	0
chrUn_gl000238	0	0	0	0
chrUn_gl000239	0	0	0	0
chrUn_gl000240	0	0	0	0
chrUn_gl000241	0	0	0	0
chrUn_gl000242	0	0	0	0
chrUn_gl000243	0	0	0	0
chrUn_gl000244	0	0	0	0
chrUn_gl000245	0	0	0	0

chrUn_gl000246	0	0	0	0
chrUn_gl000247	0	0	0	0
chrUn_gl000248	0	0	0	0
chrUn_gl000249	0	0	0	0

Coverage across reference

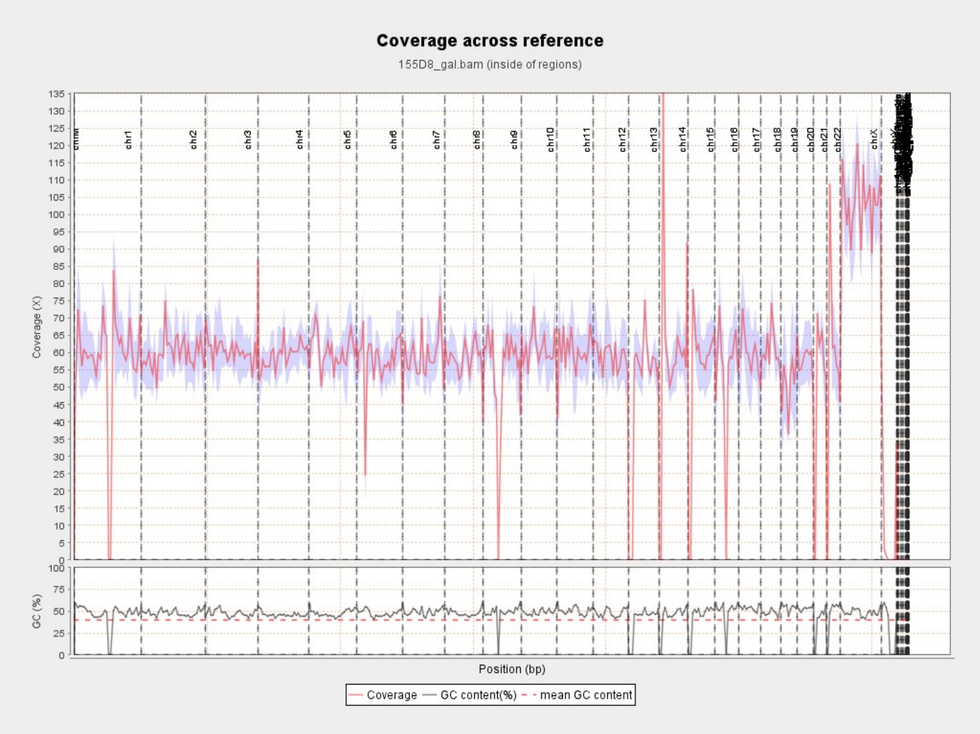

Coverage Histogram

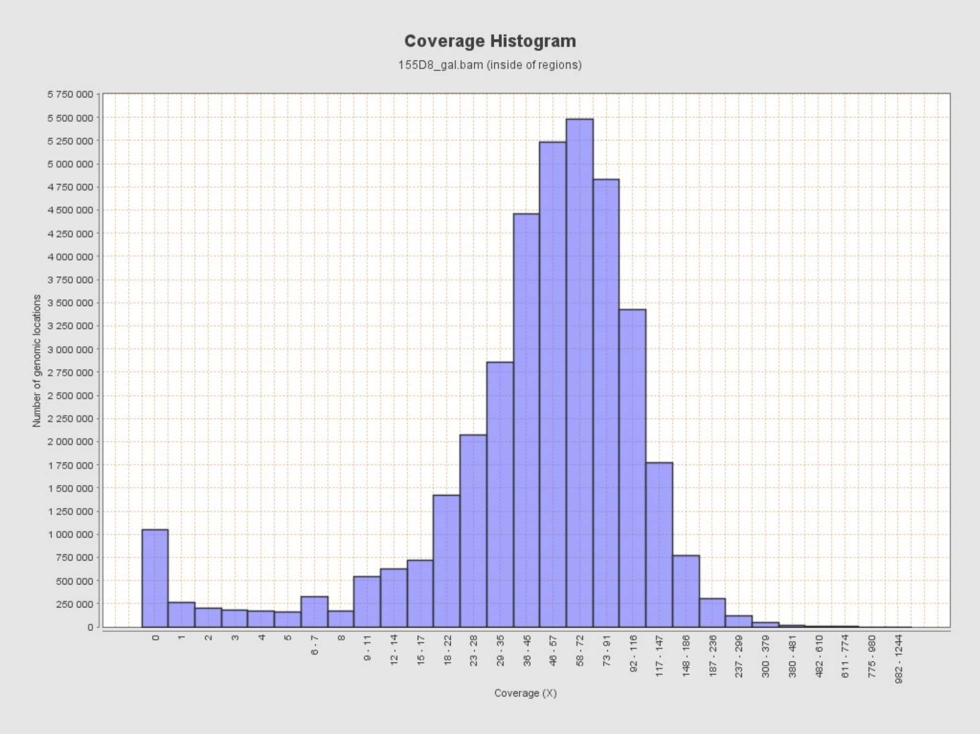

Coverage Histogram (0-50X)

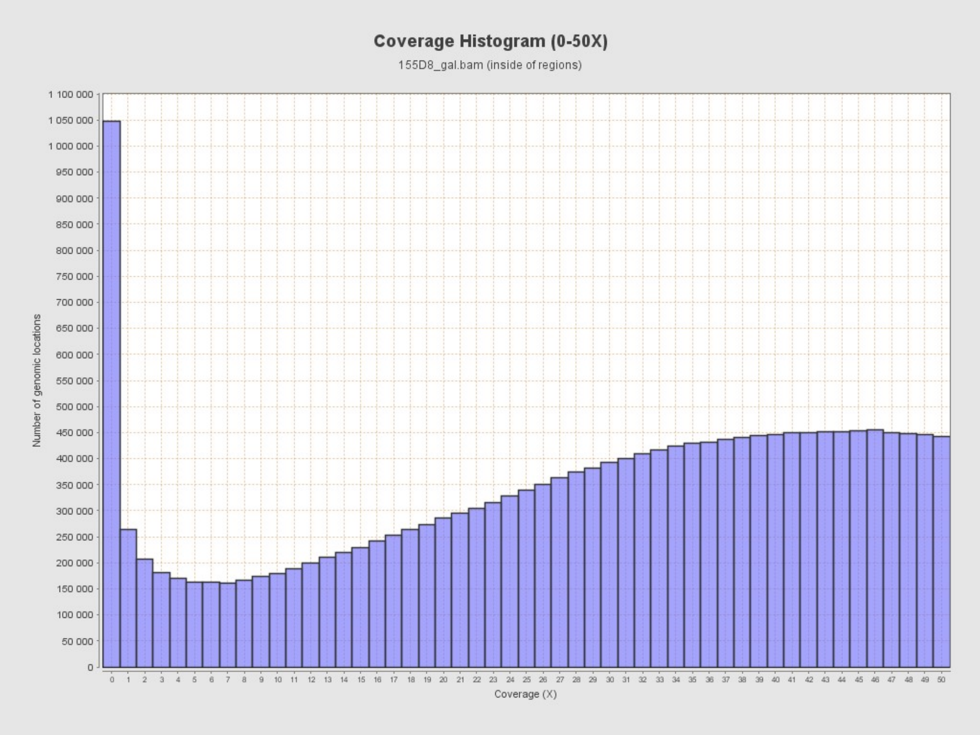

Genome Fraction Coverage

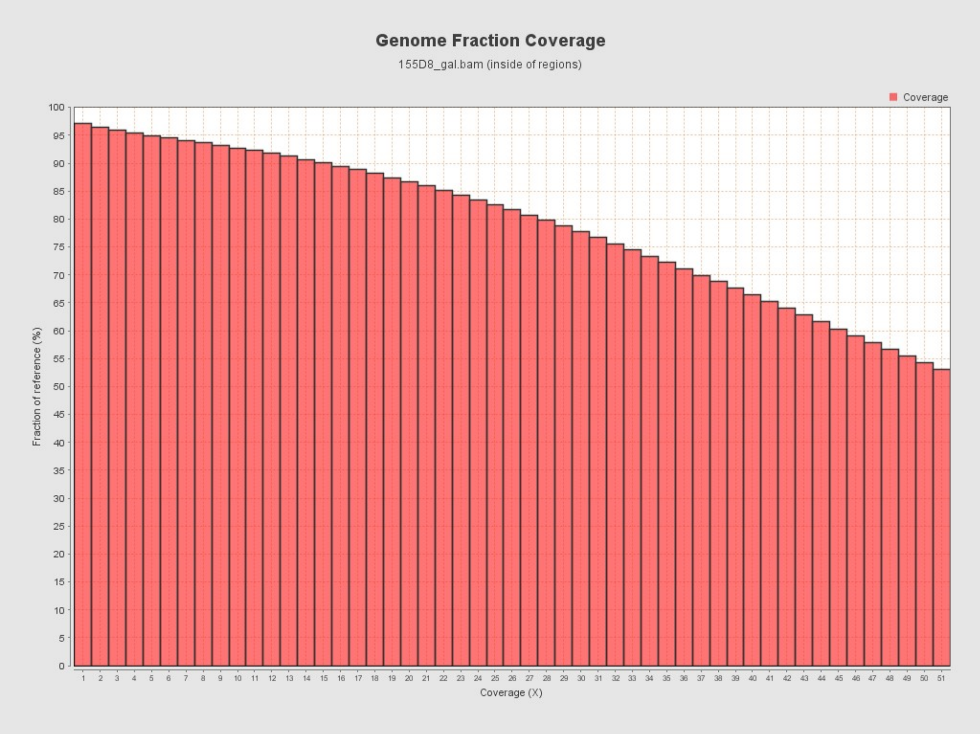

Duplication Rate Histogram

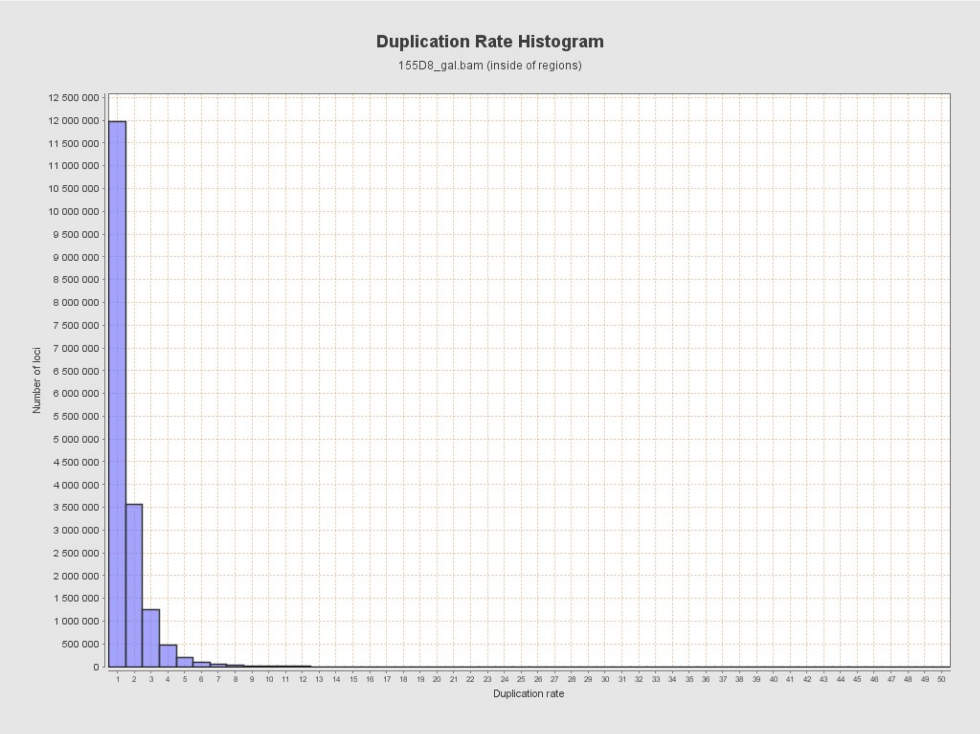

Mapped Reads Nucleotide Content

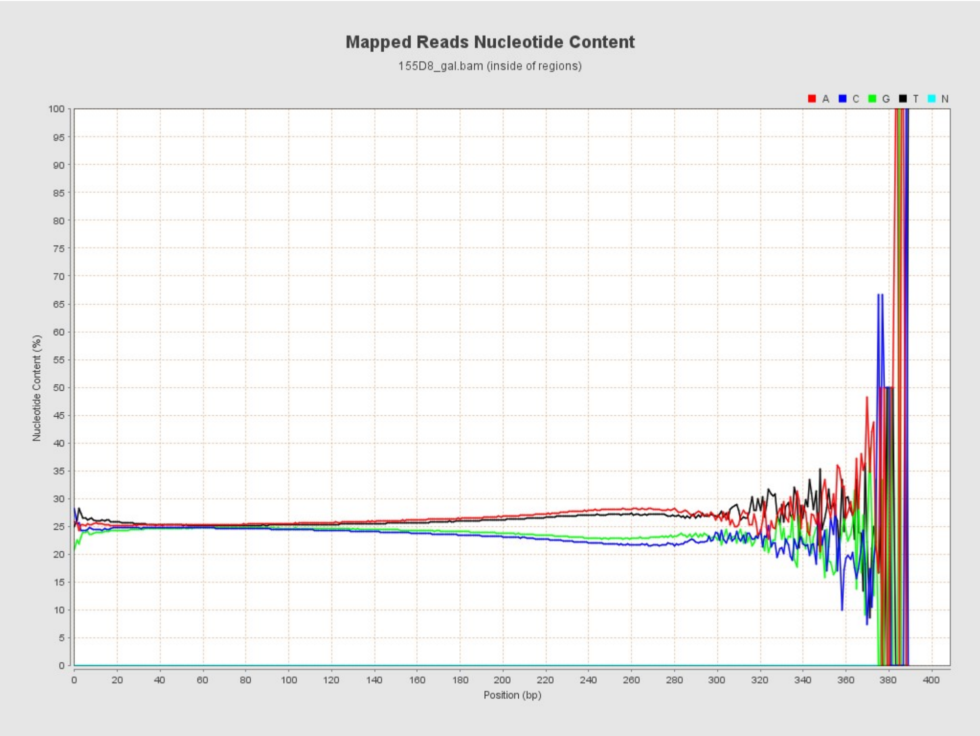

Mapped Reads GC-content Distribution

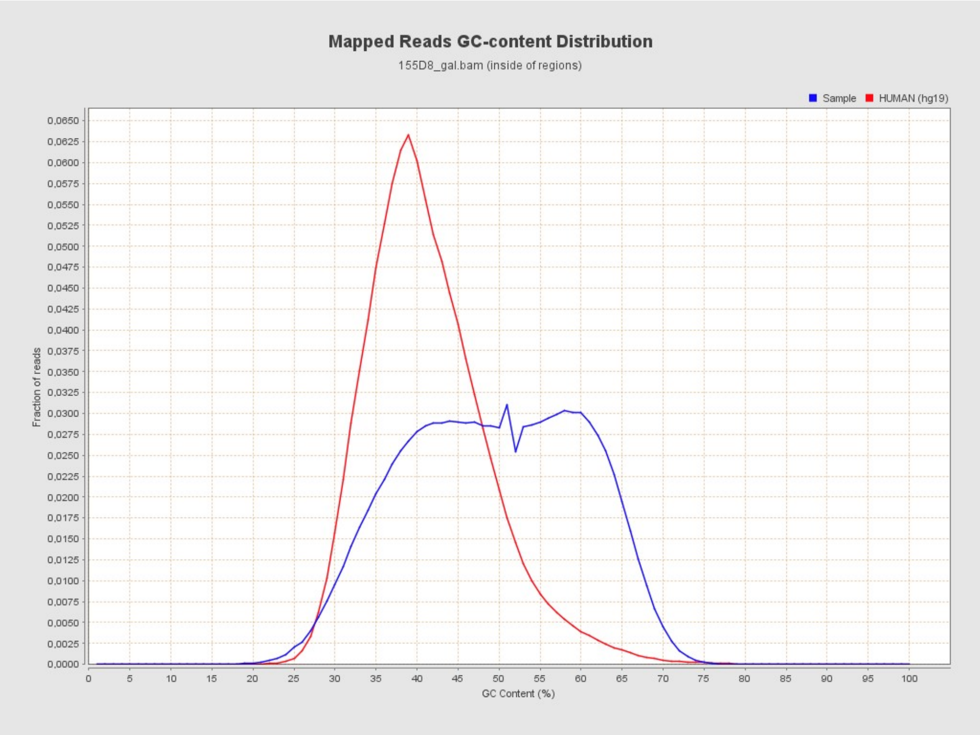

Mapped Reads Clipping Profile

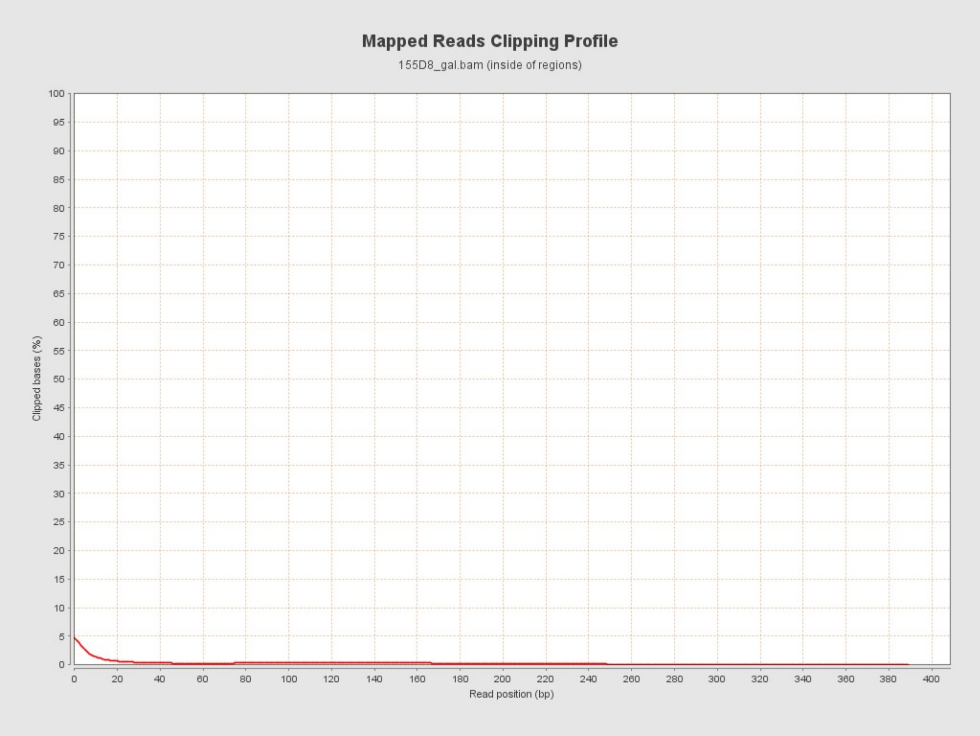

Homopolymer Indels

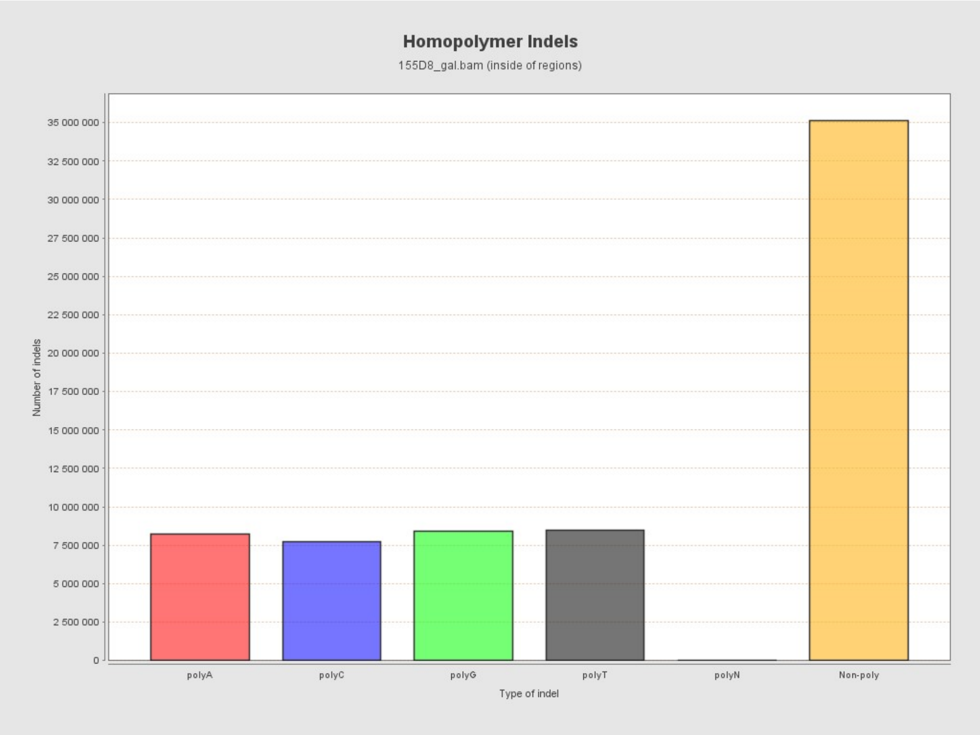

Mapping Quality Across Reference

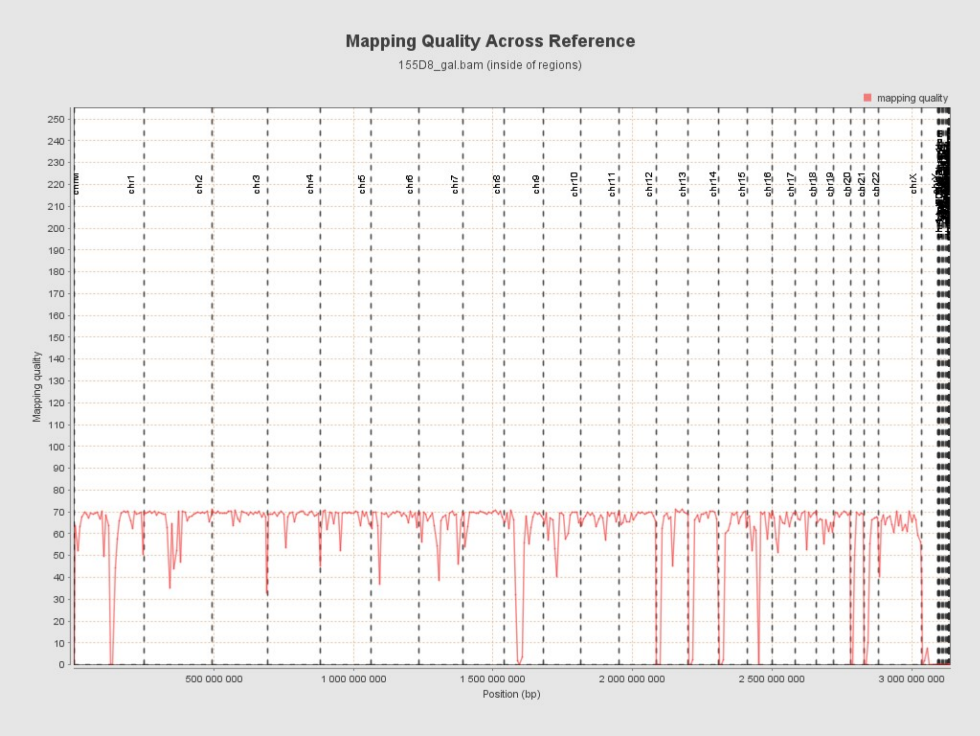

Mapping Quality Histogram

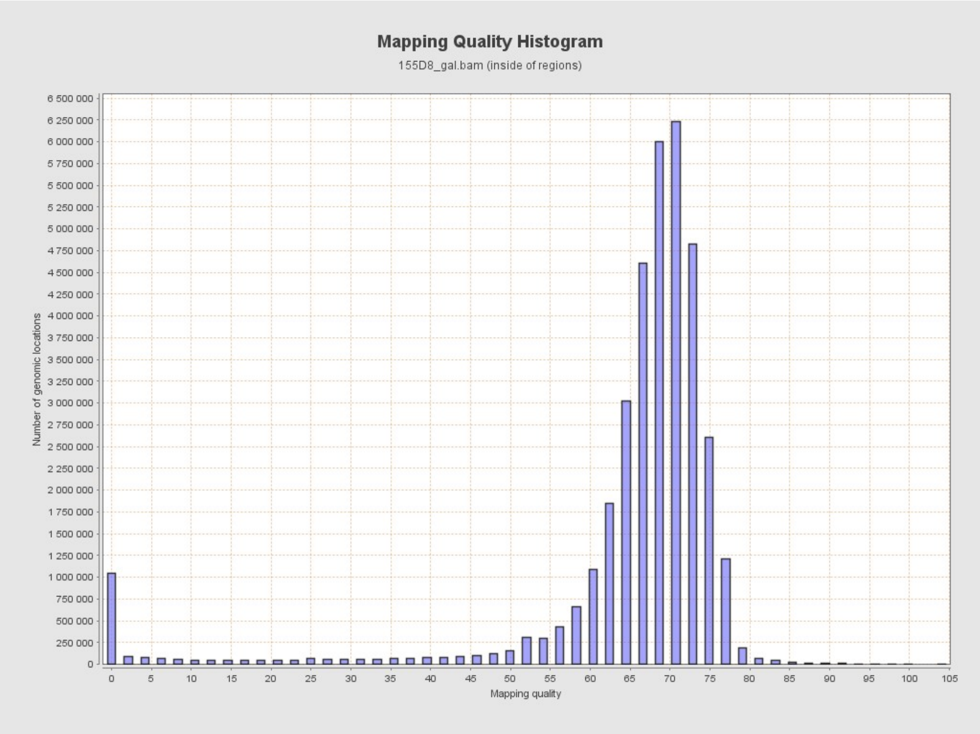

2016/11/08 09:38:29

Generated by QualiMap v.2.2.1

Qualimap Report: First tumour alignment quality metrics

QualiMap

CONTENTS

- Input data & parameters
- Summary
- Coverage across reference
- Coverage Histogram
- Coverage Histogram (0-50X)
- Genome Fraction Coverage
- Duplication Rate Histogram
- Mapped Reads Nucleotide Content
- Mapped Reads GC-content Distribution
- Mapped Reads Clipping Profile
- Homopolymer Indels
- Mapping Quality Across Reference
- Mapping Quality Histogram

Input data and parameters

QualiMap command line

qualimap bamqc -bam E:\NGS\Adenomas\Recidivs_5_31_155D8\HA05_gal.bam -gff E:\NGS \Exome_hg19_6fields.bed -os -c -nw 400 -hm 3

Alignment

Command line:	mapall -f ucsc.hg19.fasta -r HA05.fastq -i fastq -s HA05.bam -o 1 stage1 map1 stage2 map2 stage3 map3 stage4 map4
Draw chromosome limits:	yes
Analyze overlapping paired-end reads:	no
Program:	tmap (3.4.1)
Analysis date:	Tue Nov 08 10:40:21 EET 2016
Size of a homopolymer:	3
Skip duplicate alignments:	no
Number of windows:	400
BAM file:	E:\NGS\Adenomas \Recidivs_5_31_155D8\HA05_gal.bam

GFF region

Library protocol:	non-strand-specific
Outside statistics:	yes
GFF file:	E:\NGS\Exome_hg19_6fields.bed

Summary

Globals

Reference size	3 137 161 264
Number of reads	73 384 342
Mapped reads	71 615 114 / 97,59%
Unmapped reads	1 769 228 / 2,41%
Mapped paired reads	0 / 0%
Read min/max/mean length	8 / 389 / 123,47
Clipped reads	19 925 777 / 27,15%

Globals (inside of regions)

Regions size/percentage of reference	37 259 743 / 1,19%
Mapped reads	32 706 388 / 44,57%
Duplicated reads (estimated)	24 100 161 / 73,69%

Duplication rate	49,91%

ACGT Content (inside of regions)

Number/percentage of A's	581 726 650 / 22,15%
Number/percentage of C's	738 620 227 / 28,13%
Number/percentage of T's	568 923 934 / 21,67%
Number/percentage of G's	736 703 364 / 28,05%
Number/percentage of N's	0 / 0%
GC Percentage	56,18%

Coverage (inside of regions)

Mean	70,8506
Standard Deviation	210,7058

Mapping Quality (inside of regions)

Mean Mapping Quality	62,76

Mismatches and indels (inside of regions)

General error rate	4,73%
Mismatches	80 237 006
Insertions	27 628 707
Mapped reads with at least one insertion	20,81%
Deletions	17 716 669
Mapped reads with at least one deletion	16,39%
Homopolymer indels	45,07%

Chromosome stats (inside of regions)

Name	Length	Mapped bases	Mean coverage	Standard deviation
chrM	0	0	0	0
chr1	3784540	276715387	73,1173	133,8306
chr2	2784129	146072489	52,4661	89,5221
chr3	2096186	124687064	59,4828	93,5154
chr4	1419494	55259747	38,9292	89,7465
chr5	1707093	90167943	52,8196	98,0709
chr6	1939714	90427204	46,6188	124,8433
chr7	1715393	112835265	65,7781	111,9768
chr8	1223260	69866447	57,115	104,0277
chr9	1591797	116357951	73,0985	180,4604
chr10	1501994	91844406	61,1483	92,8024
chr11	2152291	164036700	76,2149	129,1423
chr12	1910362	124748953	65,3012	167,5877

chr13	706110	30614066	43,3559	84,492
chr14	1194533	82319157	68,9133	126,6364
chr15	1229578	81276655	66,1013	130,2296
chr16	1502270	140286395	93,3829	204,9517
chr17	2108732	184216850	87,3591	140,9159
chr18	566305	26803663	47,3308	90,0536
chr19	2381241	235833614	99,0381	379,24
chr20	922966	73815861	79,9768	114,0044
chr21	401819	31693229	78,8744	169,478
chr22	836825	81926950	97,9021	160,531
chrX	1493772	205745267	137,7354	703,6147
chrY	89339	2323996	26,0132	74,1616
chr1_gl000191_random	0	0	0	0
chr1_gl000192_random	0	0	0	0
chr4_ctg9_hap1	0	0	0	0
chr4_gl000193_random	0	0	0	0
chr4_gl000194_random	0	0	0	0
chr6_apd_hap1	0	0	0	0
chr6_cox_hap2	0	0	0	0
chr6_dbb_hap3	0	0	0	0
chr6_mann_hap4	0	0	0	0
chr6_mcf_hap5	0	0	0	0
chr6_qbl_hap6	0	0	0	0
chr6_ssto_hap7	0	0	0	0
chr7_gl000195_random	0	0	0	0
chr8_gl000196_random	0	0	0	0
chr8_gl000197_random	0	0	0	0
chr9_gl000198_random	0	0	0	0
chr9_gl000199_random	0	0	0	0
chr9_gl000200_random	0	0	0	0
chr9_gl000201_random	0	0	0	0
chr11_gl000202_random	0	0	0	0
chr17_ctg5_hap1	0	0	0	0
chr17_gl000203_random	0	0	0	0
chr17_gl000204_random	0	0	0	0
chr17_gl000205_random	0	0	0	0
chr17_gl000206_random	0	0	0	0
chr18_gl000207_random	0	0	0	0

chr19_gl000208_random	0	0	0	0
chr19_gl000209_random	0	0	0	0
chr21_gl000210_random	0	0	0	0
chrUn_gl000211	0	0	0	0
chrUn_gl000212	0	0	0	0
chrUn_gl000213	0	0	0	0
chrUn_gl000214	0	0	0	0
chrUn_gl000215	0	0	0	0
chrUn_gl000216	0	0	0	0
chrUn_gl000217	0	0	0	0
chrUn_gl000218	0	0	0	0
chrUn_gl000219	0	0	0	0
chrUn_gl000220	0	0	0	0
chrUn_gl000221	0	0	0	0
chrUn_gl000222	0	0	0	0
chrUn_gl000223	0	0	0	0
chrUn_gl000224	0	0	0	0
chrUn_gl000225	0	0	0	0
chrUn_gl000226	0	0	0	0
chrUn_gl000227	0	0	0	0
chrUn_gl000228	0	0	0	0
chrUn_gl000229	0	0	0	0
chrUn_gl000230	0	0	0	0
chrUn_gl000231	0	0	0	0
chrUn_gl000232	0	0	0	0
chrUn_gl000233	0	0	0	0
chrUn_gl000234	0	0	0	0
chrUn_gl000235	0	0	0	0
chrUn_gl000236	0	0	0	0
chrUn_gl000237	0	0	0	0
chrUn_gl000238	0	0	0	0
chrUn_gl000239	0	0	0	0
chrUn_gl000240	0	0	0	0
chrUn_gl000241	0	0	0	0
chrUn_gl000242	0	0	0	0
chrUn_gl000243	0	0	0	0
chrUn_gl000244	0	0	0	0
chrUn_gl000245	0	0	0	0

chrUn_gl000246	0	0	0	0
chrUn_gl000247	0	0	0	0
chrUn_gl000248	0	0	0	0
chrUn_gl000249	0	0	0	0

Coverage across reference

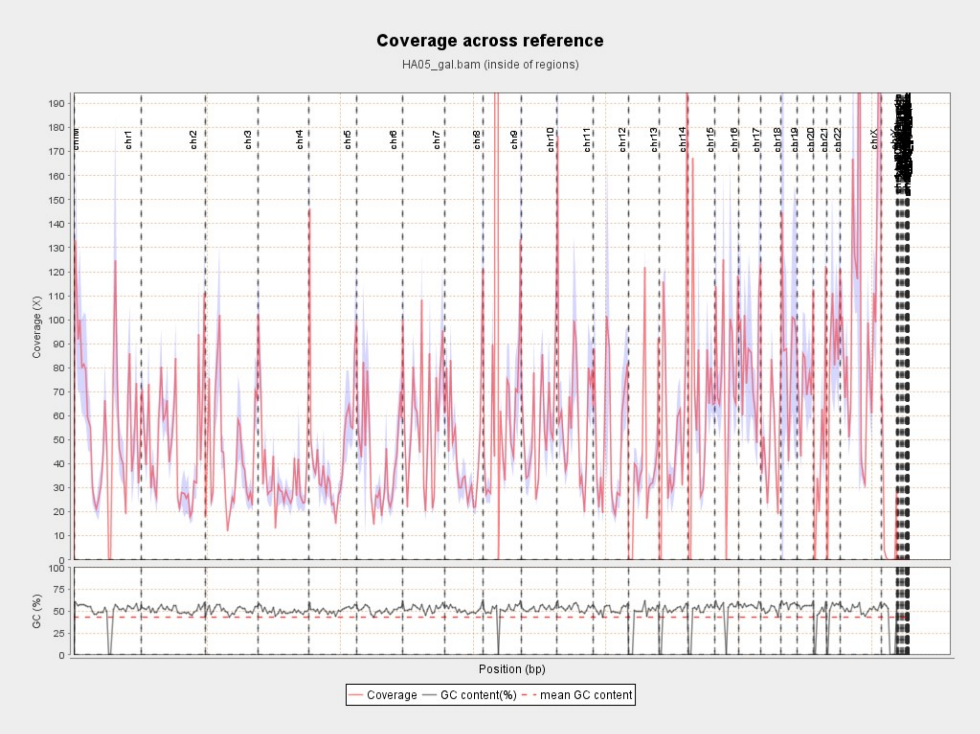

Coverage Histogram

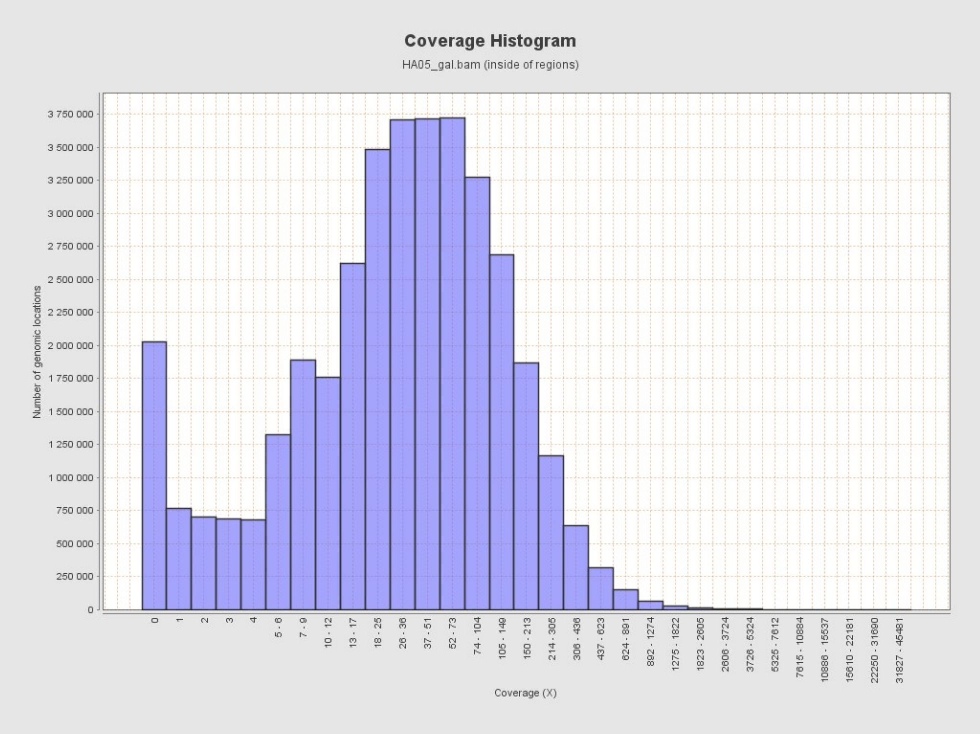

Coverage Histogram (0-50X)

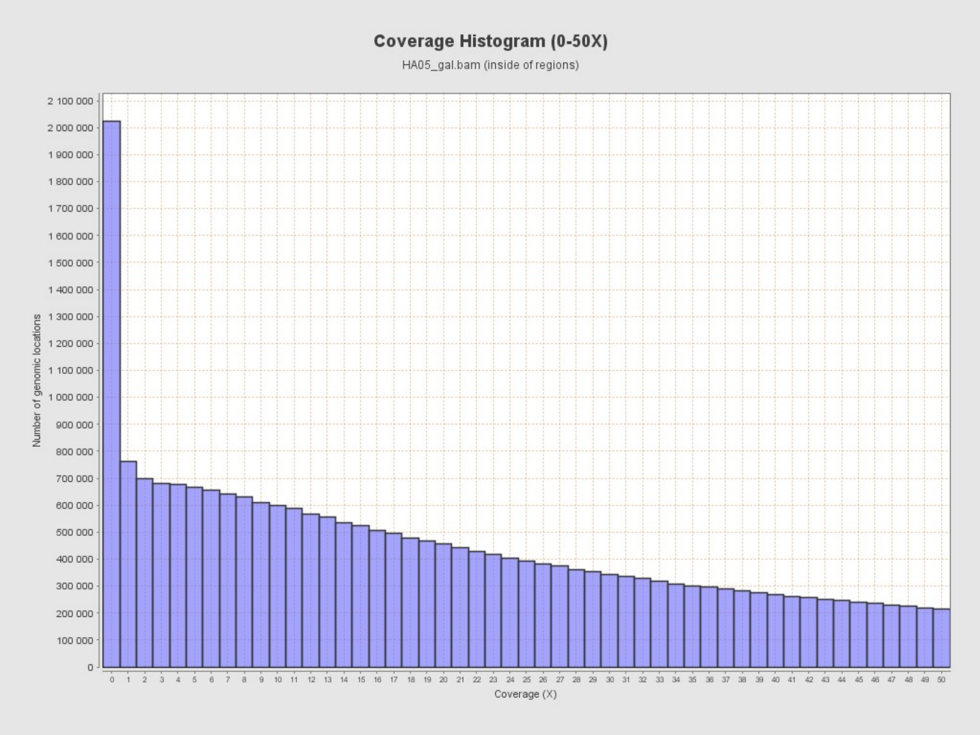

Genome Fraction Coverage

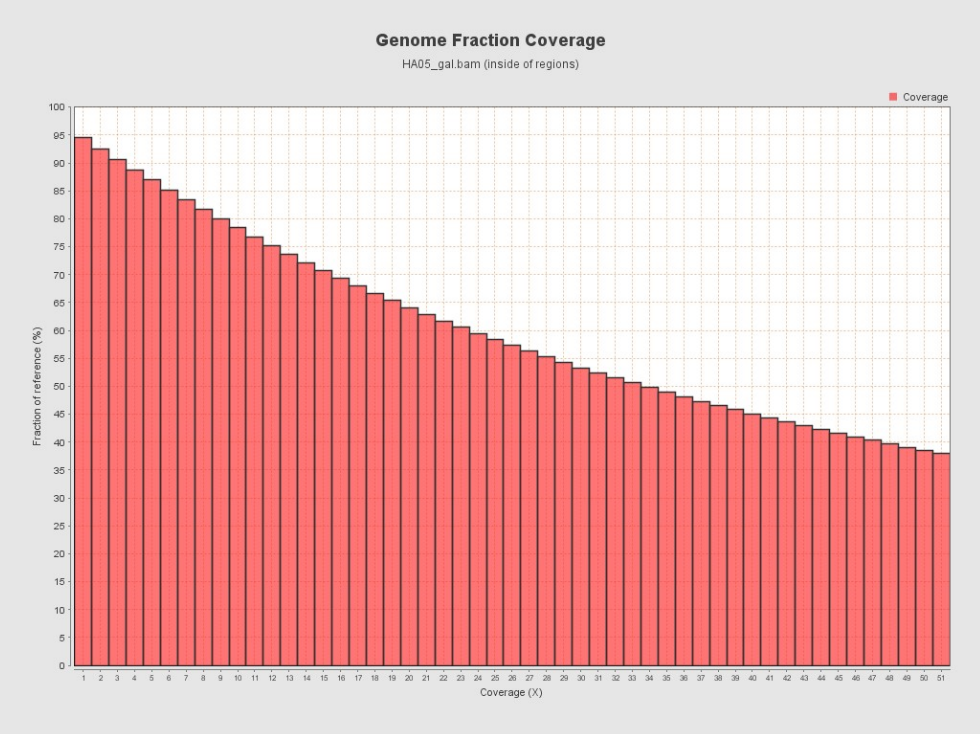

Duplication Rate Histogram

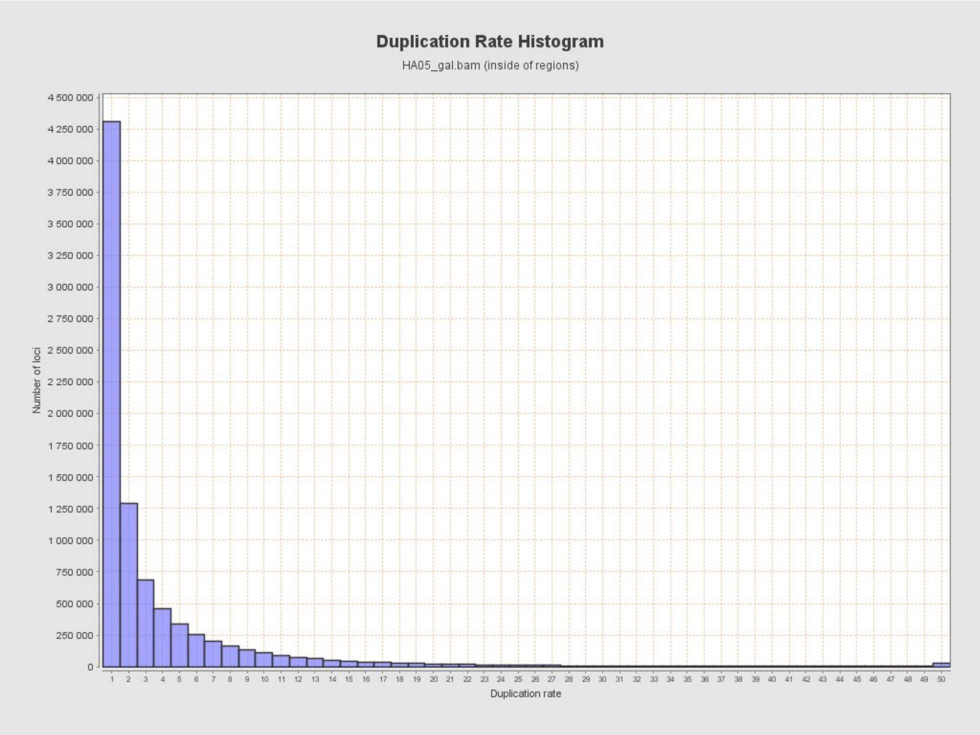

Mapped Reads Nucleotide Content

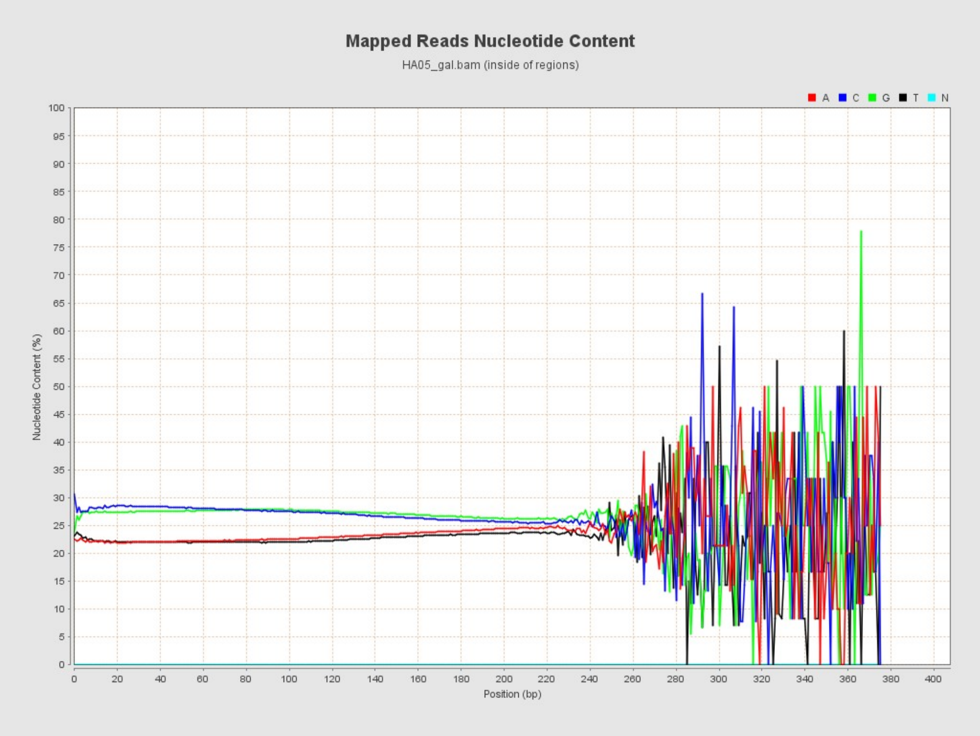

Mapped Reads GC-content Distribution

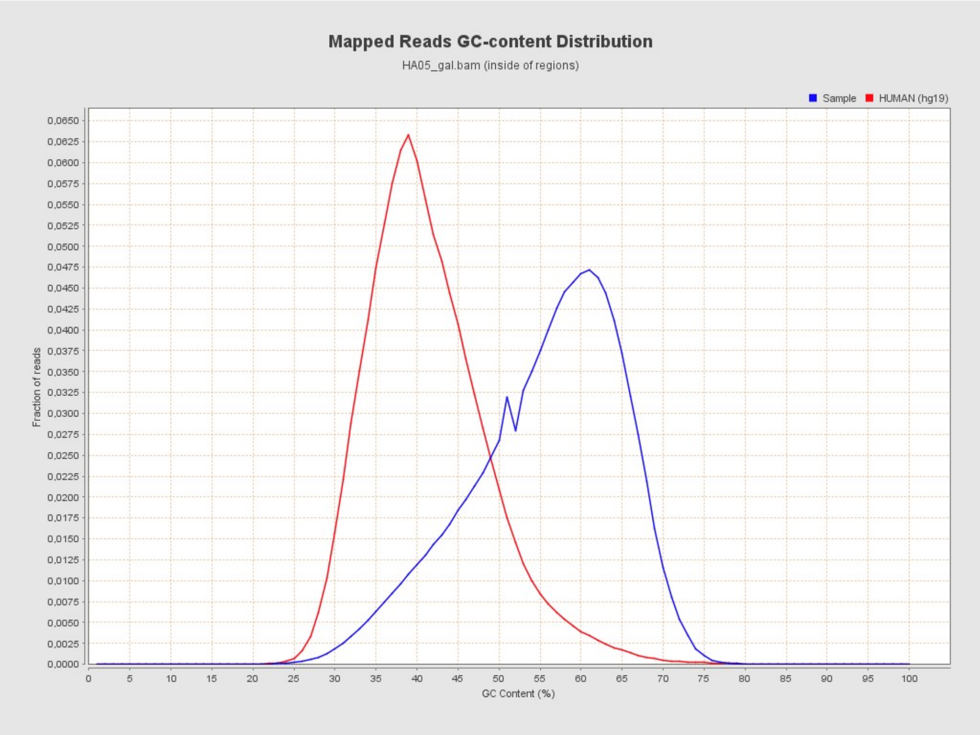

Mapped Reads Clipping Profile

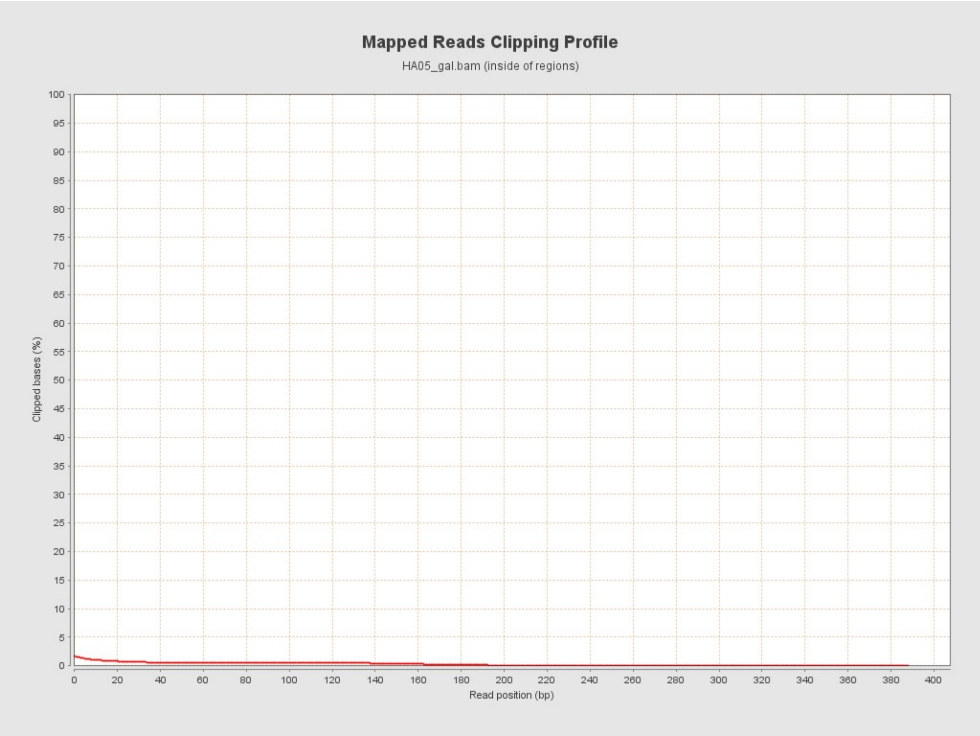

Homopolymer Indels

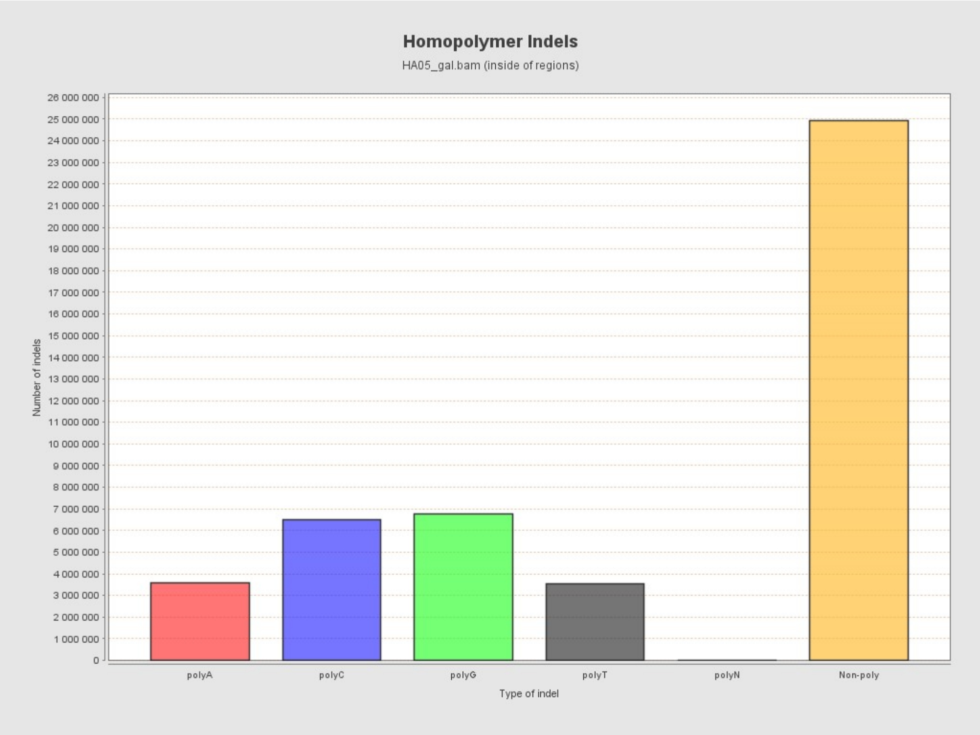

Mapping Quality Across Reference

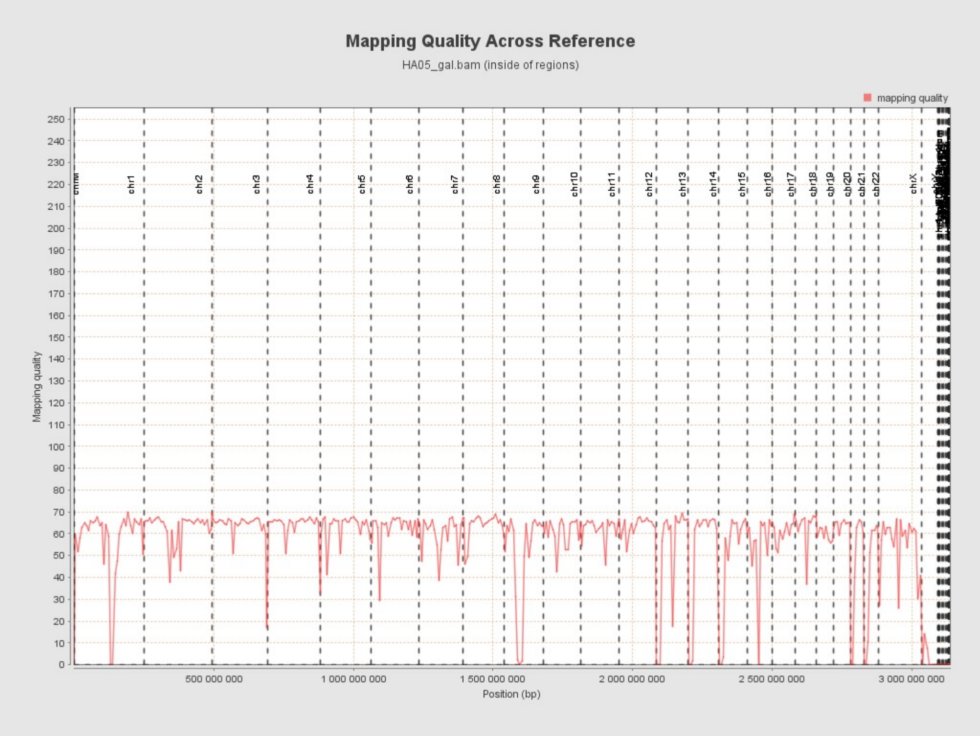

Mapping Quality Histogram

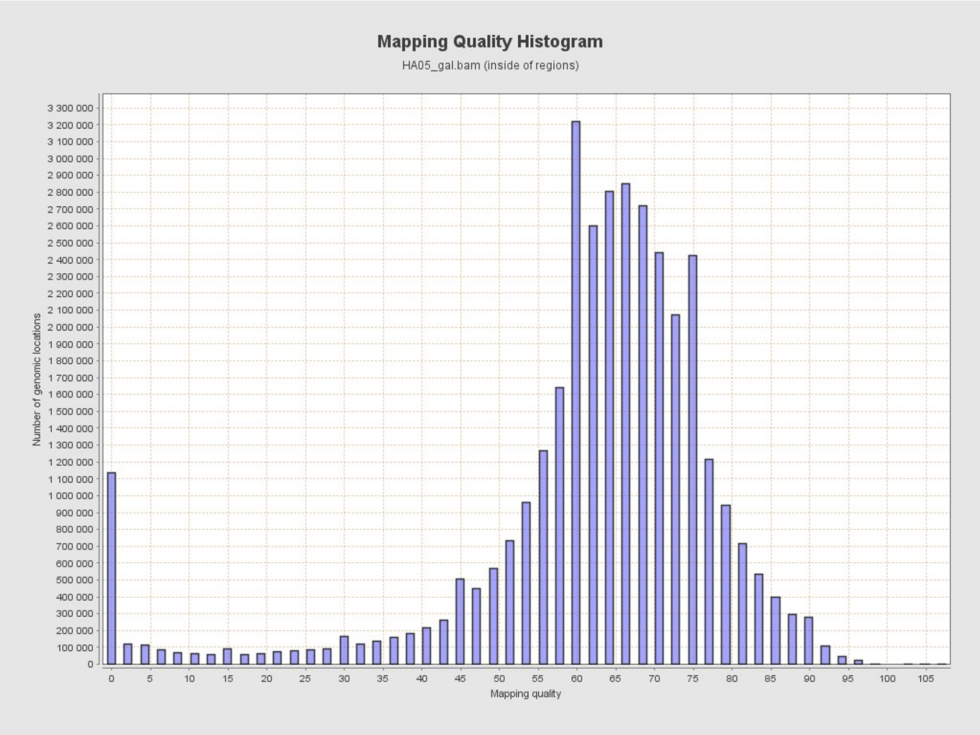

2016/11/08 12:45:05

Generated by QualiMap v.2.2.1

Qualimap Report: Second tumour DNA alignment quality metrics

CONTENTS

- Input data & parameters
- Summary
- Coverage across reference
- Coverage Histogram
- Coverage Histogram (0-50X)
- Genome Fraction Coverage
- Duplication Rate Histogram
- Mapped Reads Nucleotide Content
- Mapped Reads GC-content Distribution
- Mapped Reads Clipping Profile
- Homopolymer Indels
- Mapping Quality Across Reference
- Mapping Quality Histogram

Input data and parameters

QualiMap command line

```
qualimap bamqc -bam E:\NGS\Adenomas\Recidivs_5_31_155D8\HA31_gal.bam -gff E:\NGS\Exome_hg19_6fields.bed -os -c -nw 400 -hm 3
```

Alignment

Command line:	mapall -f ucsc.hg19.fasta -r HA31.fastq -i fastq -s HA31.bam -o 1 stage1 map1 stage2 map2 stage3 map3 stage4 map4
Draw chromosome limits:	yes
Analyze overlapping paired-end reads:	no
Program:	tmap (3.4.1)
Analysis date:	Tue Nov 08 09:59:32 EET 2016
Size of a homopolymer:	3
Skip duplicate alignments:	no
Number of windows:	400
BAM file:	E:\NGS\Adenomas \Recidivs_5_31_155D8\HA31_gal.bam

GFF region

Library protocol:	non-strand-specific
Outside statistics:	yes
GFF file:	E:\NGS\Exome_hg19_6fields.bed

Summary

Globals

Reference size	3 137 161 264
Number of reads	90 113 032
Mapped reads	87 751 327 / 97,38%
Unmapped reads	2 361 705 / 2,62%
Mapped paired reads	0 / 0%
Read min/max/mean length	8 / 388 / 123,36
Clipped reads	23 325 266 / 25,88%

Globals (inside of regions)

Regions size/percentage of reference	37 259 743 / 1,19%
Mapped reads	38 664 636 / 42,91%
Duplicated reads (estimated)	25 157 057 / 65,06%

Duplication rate	50,48%

ACGT Content (inside of regions)

Number/percentage of A's	713 283 702 / 22,68%
Number/percentage of C's	865 579 777 / 27,53%
Number/percentage of T's	699 162 525 / 22,24%
Number/percentage of G's	866 304 823 / 27,55%
Number/percentage of N's	0 / 0%
GC Percentage	55,08%

Coverage (inside of regions)

Mean	84,8354
Standard Deviation	273,9178

Mapping Quality (inside of regions)

Mean Mapping Quality	64

Mismatches and indels (inside of regions)

General error rate	4,44%
Mismatches	89 257 377
Insertions	33 263 461
Mapped reads with at least one insertion	20,19%
Deletions	21 418 865
Mapped reads with at least one deletion	16,08%
Homopolymer indels	45,2%

Chromosome stats (inside of regions)

Name	Length	Mapped bases	Mean coverage	Standard deviation
chrM	0	0	0	0
chr1	3784540	329356270	87,0268	167,8278
chr2	2784129	178708878	64,1884	102,2274
chr3	2096186	149504515	71,3222	104,9439
chr4	1419494	72217121	50,8753	106,7254
chr5	1707093	111212663	65,1474	116,956
chr6	1939714	118480442	61,0814	217,8677
chr7	1715393	133907003	78,062	129,7076
chr8	1223260	83567326	68,3153	113,2658
chr9	1591797	139748348	87,7928	325,4959
chr10	1501994	109990653	73,2298	97,3256
chr11	2152291	196884234	91,4766	171,6482
chr12	1910362	152223482	79,6831	234,9356

chr13	706110	39099101	55,3725	99,926
chr14	1194533	100402338	84,0515	149,8884
chr15	1229578	96823102	78,745	174,2551
chr16	1502270	161393152	107,4329	261,6091
chr17	2108732	211280718	100,1933	160,5545
chr18	566305	33163852	58,5618	92,8592
chr19	2381241	278679429	117,0312	491,7239
chr20	922966	83895987	90,8982	119,664
chr21	401819	40641785	101,1445	357,9042
chr22	836825	93564831	111,8093	222,8602
chrX	1493772	243391489	162,9375	885,9261
chrY	89339	2809730	31,4502	97,8118
chr1_gl000191_random	0	0	0	0
chr1_gl000192_random	0	0	0	0
chr4_ctg9_hap1	0	0	0	0
chr4_gl000193_random	0	0	0	0
chr4_gl000194_random	0	0	0	0
chr6_apd_hap1	0	0	0	0
chr6_cox_hap2	0	0	0	0
chr6_dbb_hap3	0	0	0	0
chr6_mann_hap4	0	0	0	0
chr6_mcf_hap5	0	0	0	0
chr6_qbl_hap6	0	0	0	0
chr6_ssto_hap7	0	0	0	0
chr7_gl000195_random	0	0	0	0
chr8_gl000196_random	0	0	0	0
chr8_gl000197_random	0	0	0	0
chr9_gl000198_random	0	0	0	0
chr9_gl000199_random	0	0	0	0
chr9_gl000200_random	0	0	0	0
chr9_gl000201_random	0	0	0	0
chr11_gl000202_random	0	0	0	0
chr17_ctg5_hap1	0	0	0	0
chr17_gl000203_random	0	0	0	0
chr17_gl000204_random	0	0	0	0
chr17_gl000205_random	0	0	0	0
chr17_gl000206_random	0	0	0	0
chr18_gl000207_random	0	0	0	0

chr19_gl000208_random	0	0	0	0
chr19_gl000209_random	0	0	0	0
chr21_gl000210_random	0	0	0	0
chrUn_gl000211	0	0	0	0
chrUn_gl000212	0	0	0	0
chrUn_gl000213	0	0	0	0
chrUn_gl000214	0	0	0	0
chrUn_gl000215	0	0	0	0
chrUn_gl000216	0	0	0	0
chrUn_gl000217	0	0	0	0
chrUn_gl000218	0	0	0	0
chrUn_gl000219	0	0	0	0
chrUn_gl000220	0	0	0	0
chrUn_gl000221	0	0	0	0
chrUn_gl000222	0	0	0	0
chrUn_gl000223	0	0	0	0
chrUn_gl000224	0	0	0	0
chrUn_gl000225	0	0	0	0
chrUn_gl000226	0	0	0	0
chrUn_gl000227	0	0	0	0
chrUn_gl000228	0	0	0	0
chrUn_gl000229	0	0	0	0
chrUn_gl000230	0	0	0	0
chrUn_gl000231	0	0	0	0
chrUn_gl000232	0	0	0	0
chrUn_gl000233	0	0	0	0
chrUn_gl000234	0	0	0	0
chrUn_gl000235	0	0	0	0
chrUn_gl000236	0	0	0	0
chrUn_gl000237	0	0	0	0
chrUn_gl000238	0	0	0	0
chrUn_gl000239	0	0	0	0
chrUn_gl000240	0	0	0	0
chrUn_gl000241	0	0	0	0
chrUn_gl000242	0	0	0	0
chrUn_gl000243	0	0	0	0
chrUn_gl000244	0	0	0	0
chrUn_gl000245	0	0	0	0

chrUn_gl000246	0	0	0	0
chrUn_gl000247	0	0	0	0
chrUn_gl000248	0	0	0	0
chrUn_gl000249	0	0	0	0

Coverage across reference

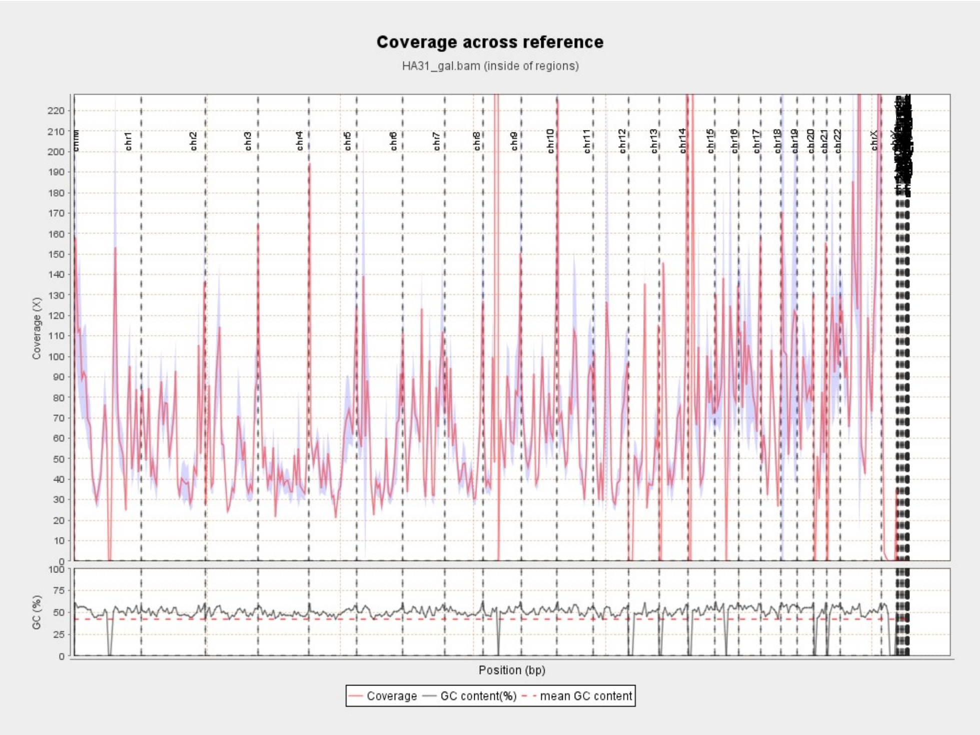

Coverage Histogram

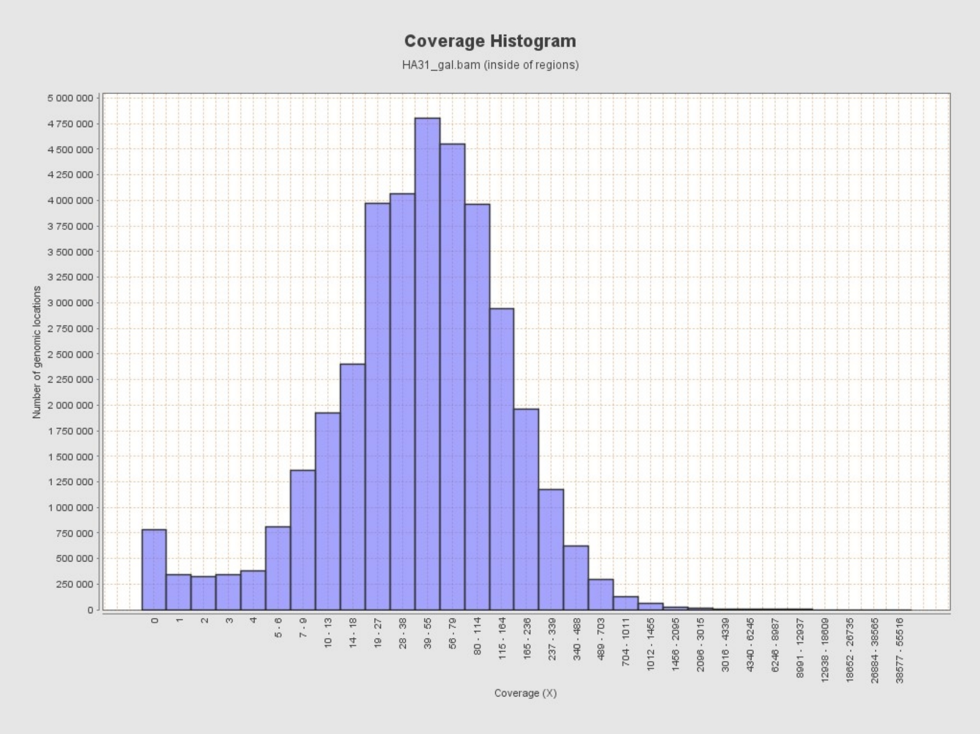

Coverage Histogram (0-50X)

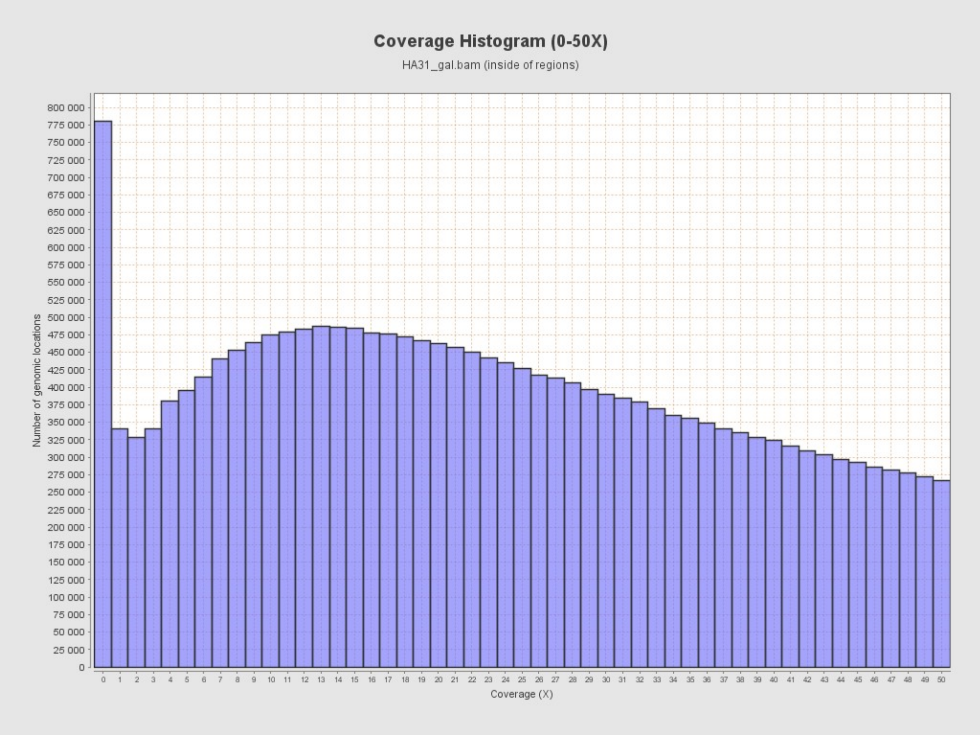

Genome Fraction Coverage

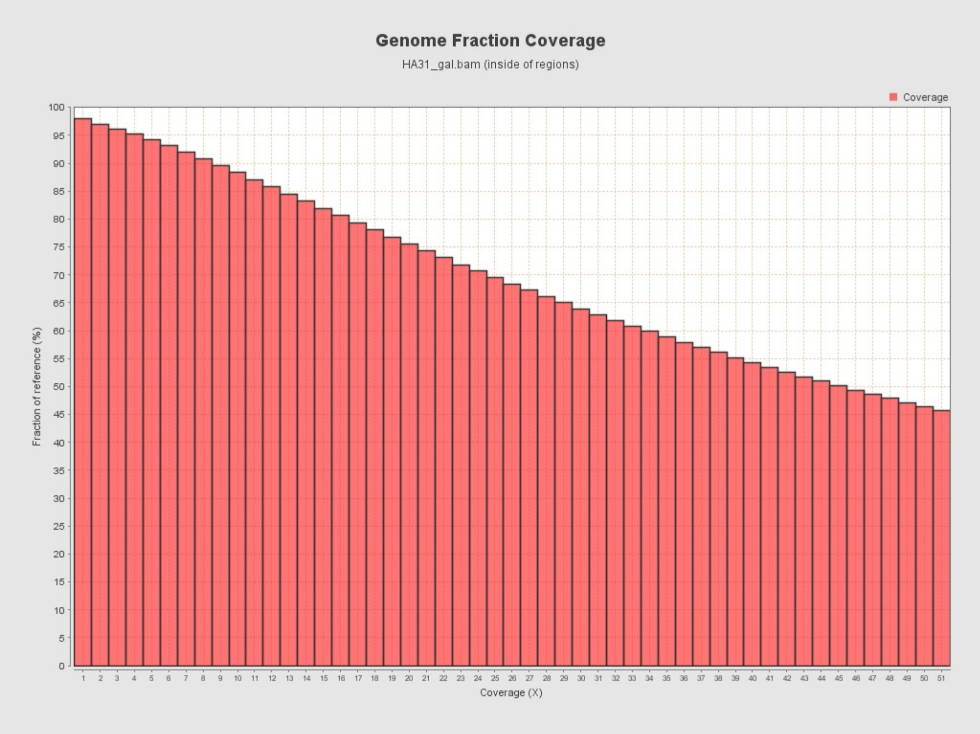

Duplication Rate Histogram

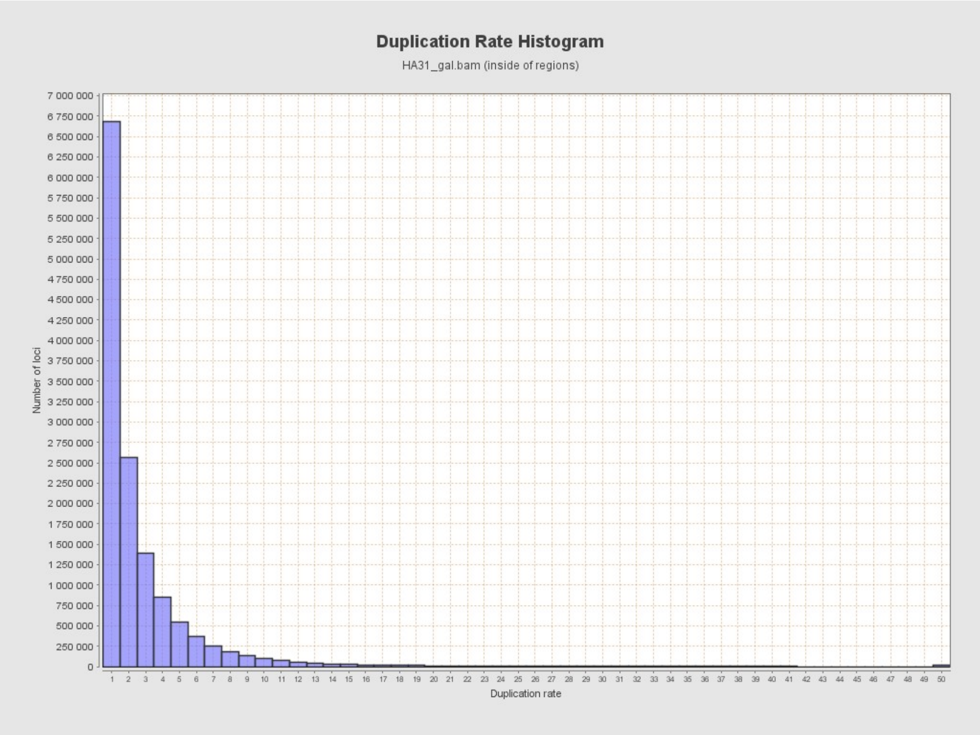

Mapped Reads Nucleotide Content

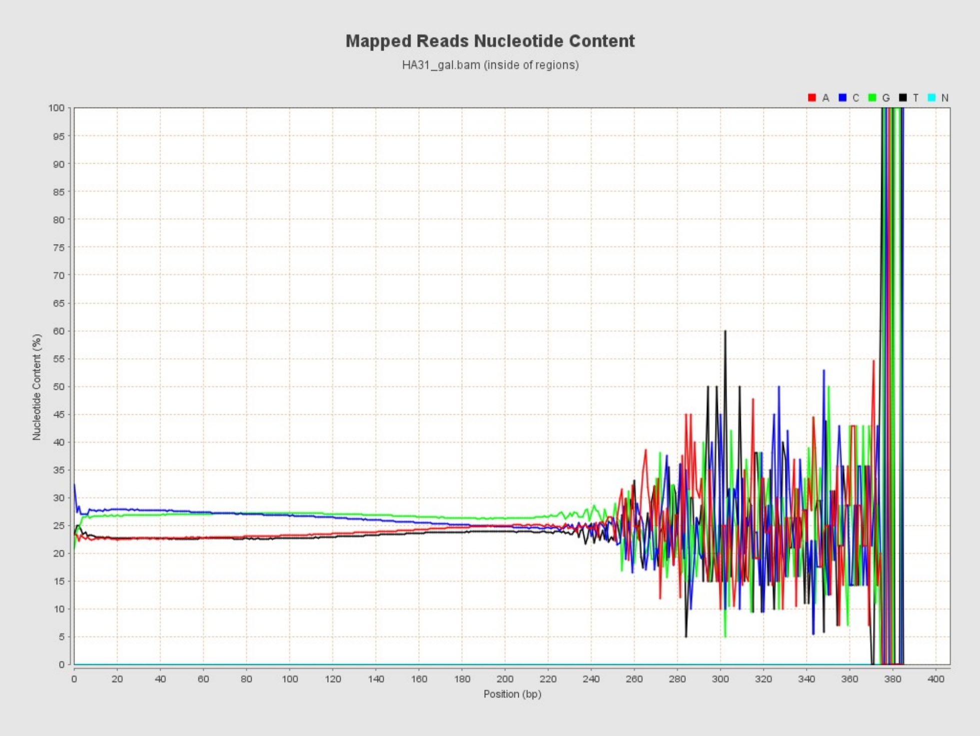

Mapped Reads GC-content Distribution

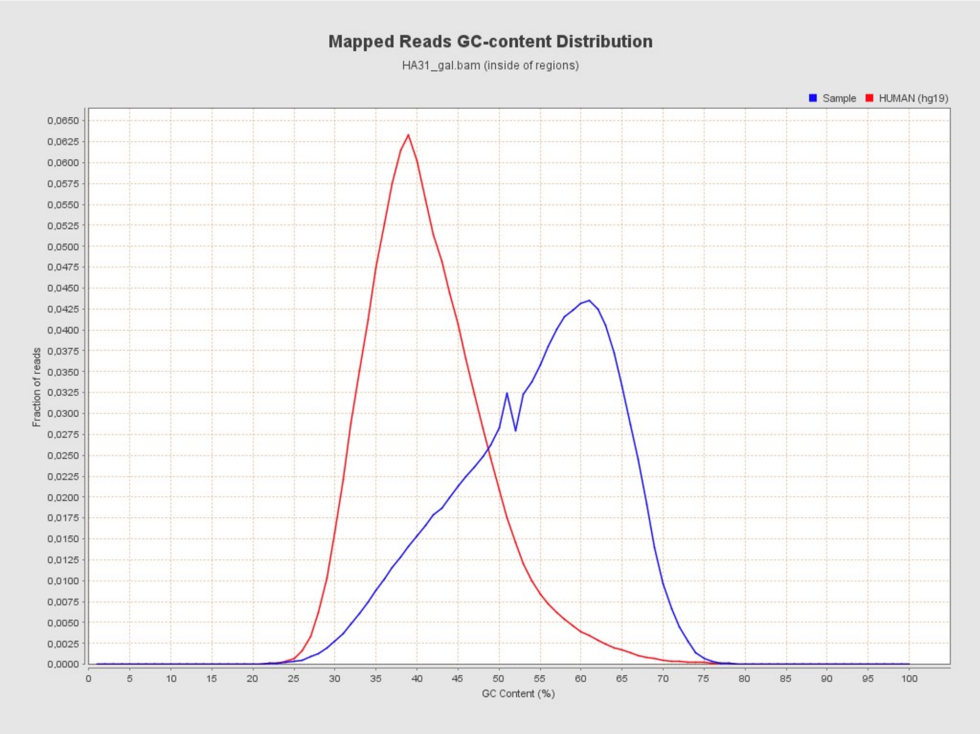

Mapped Reads Clipping Profile

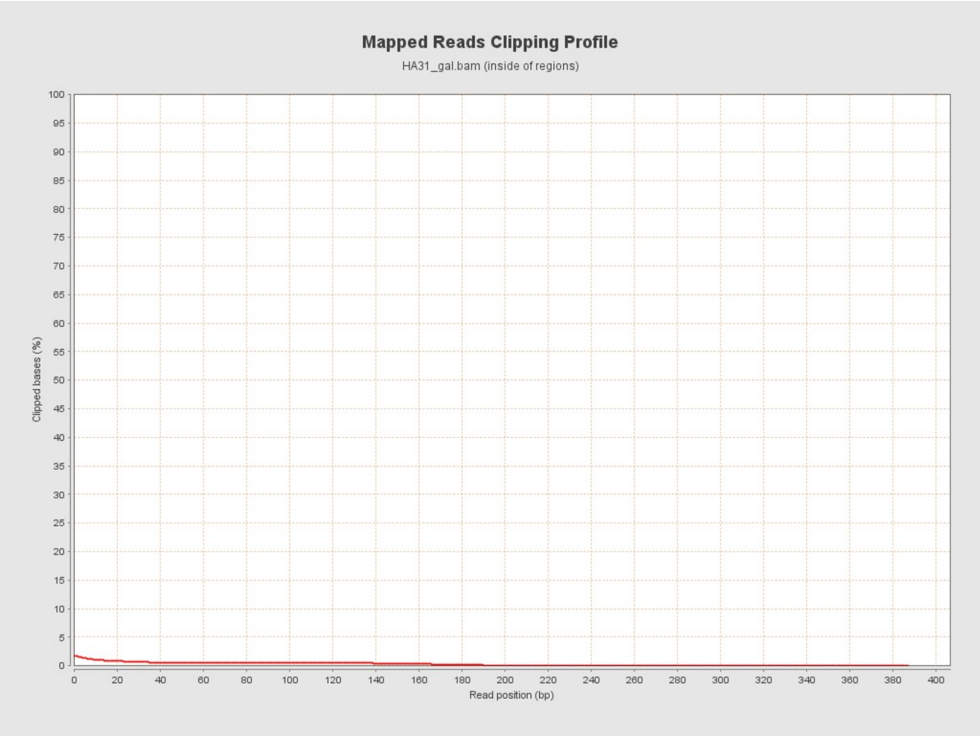

Homopolymer Indels

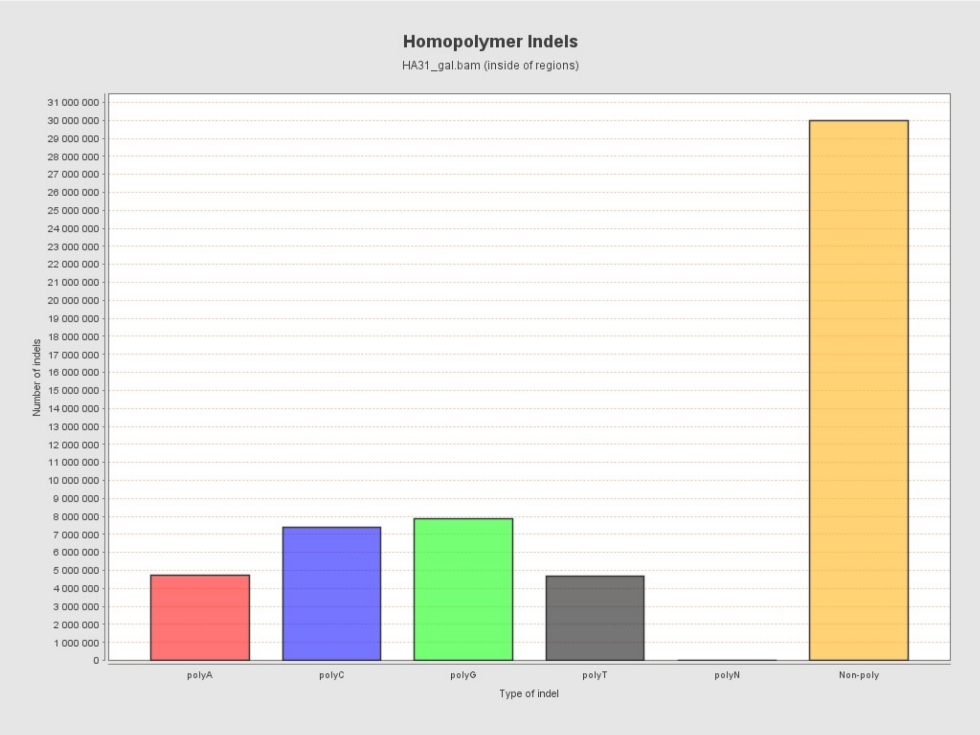

Mapping Quality Across Reference

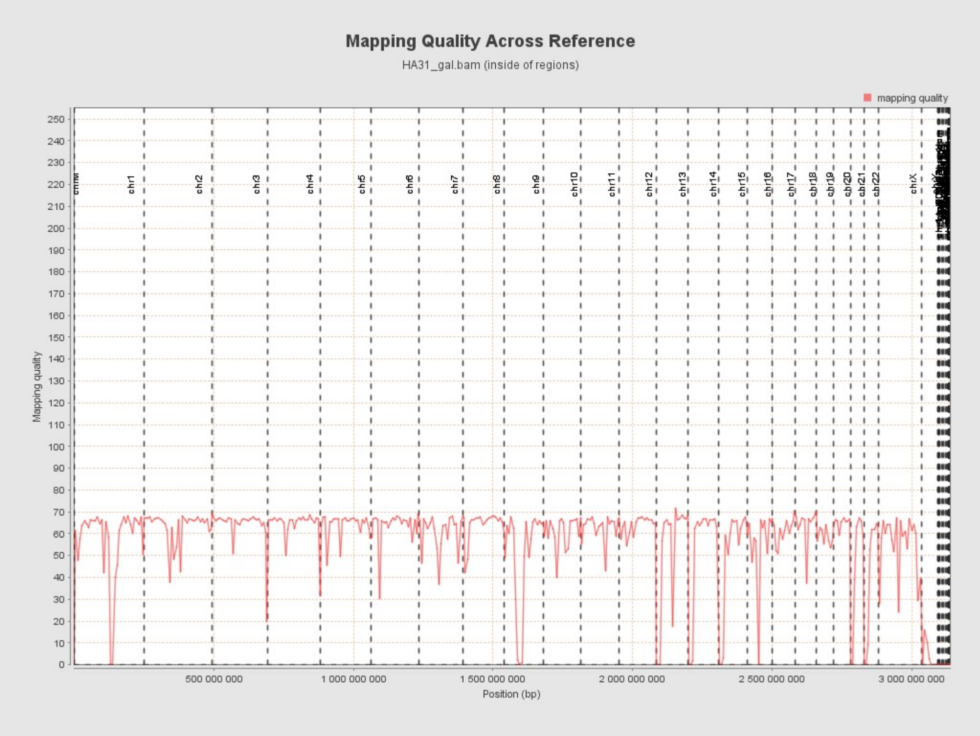

Mapping Quality Histogram

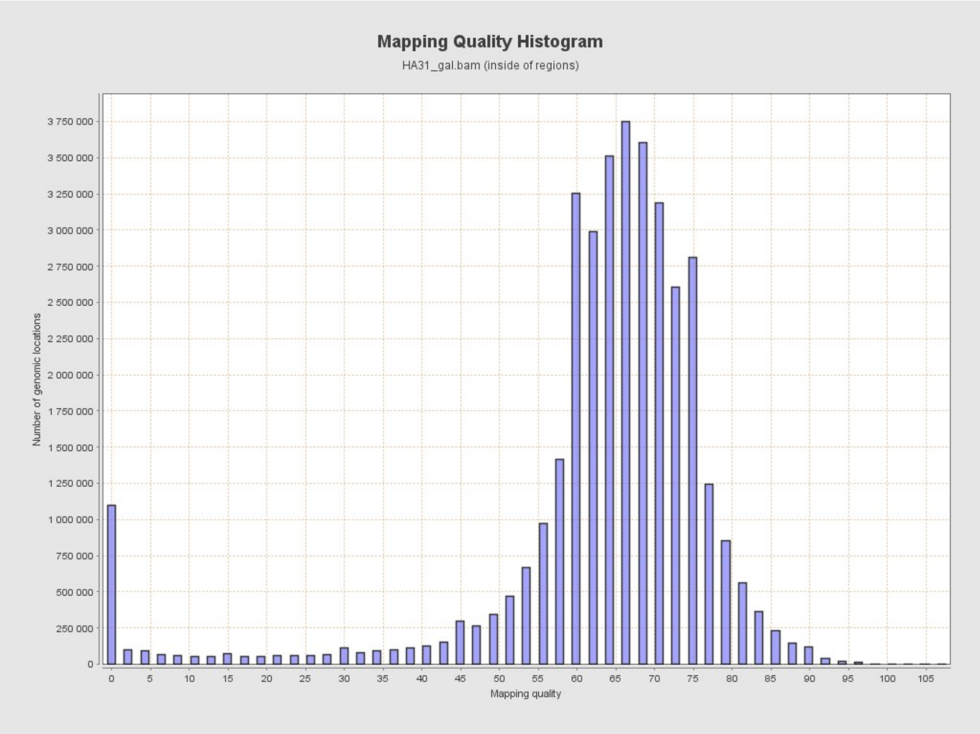

2016/11/08 10:22:17

Generated by QualiMap v.2.2.1
